# Frequency and time domain ^19^F ENDOR spectroscopy: role of nuclear dipolar couplings to determine distance distributions†

**DOI:** 10.1039/d4cp04443f

**Published:** 2024-12-12

**Authors:** Annemarie Kehl, Lucca Sielaff, Laura Remmel, Maya L. Rämisch, Marina Bennati, Andreas Meyer

**Affiliations:** a Research Group ESR Spectroscopy, Max Planck Institute for Multidisciplinary Sciences Am Fassberg 11 Göttingen Germany annemarie.kehl@mpinat.mpg.de marina.bennati@mpinat.mpg.de andreas.meyer@mpinat.mpg.de; b Georg-August-Universität Göttingen, Institute of Physical Chemistry Tammannstr. 6 Göttingen Germany

## Abstract

^19^F electron-nuclear double resonance (ENDOR) spectroscopy is emerging as a method of choice to determine molecular distances in biomolecules in the angstrom to nanometer range. However, line broadening mechanisms in ^19^F ENDOR spectra can obscure the detected spin-dipolar coupling that encodes the distance information, thus limiting the resolution and accessible distance range. So far, the origin of these mechanisms has not been understood. Here, we employ a combined approach of rational molecular design, frequency and time domain ENDOR methods as well as quantum mechanical spin dynamics simulations to analyze these mechanisms. We present the first application of Fourier transform ENDOR to remove power broadening and measure *T*_2n_ of the ^19^F nucleus. We identify nuclear dipolar couplings between the fluorine and protons up to 14 kHz as a major source of spectral broadening. When removing these interactions by H/D exchange, an unprecedented spectral width of 9 kHz was observed suggesting that, generally, the accessible distance range can be extended. In a spin labeled RNA duplex we were able to predict the spectral ENDOR line width, which in turn enabled us to extract a distance distribution. This study represents a first step towards a quantitative determination of distance distributions in biomolecules from ^19^F ENDOR.

## Abstract 2

We investigate the origins of the spectral line width parameter in ^19^F electron nuclear double resonance, which is of key importance for simulating spectra of disordered systems.

## Graphical abstract

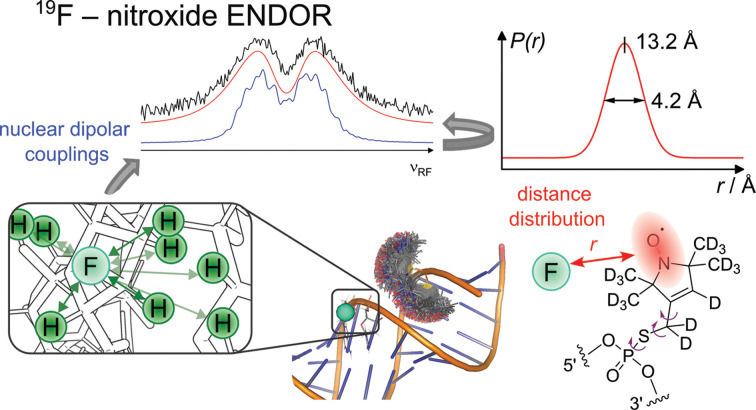

## 1 Introduction

Determination of structural information is a crucial step towards understanding the function of biological macromolecules such as proteins and nucleic acids. Paramagnetic centers, either natural ones or chemically introduced spin labels, can be investigated by electron paramagnetic resonance (EPR/ESR) spectroscopy.^1–4^ EPR provides sensitivity to enable measurements at submicromolar concentrations or in cells.^5–7^ Furthermore, several EPR-based methods for measuring distances at the scale of nanometers exist. A distance range of about 15–80 Å can be accessed by pulsed dipolar spectroscopy (PDS), where 4-pulse double electron–electron resonance (DEER, aka pulsed electron–electron double resonance, PELDOR) is the most widespread method.^8–10^ Considering the growing recognition that structural heterogeneity and intrinsic disorder play important roles in protein biochemistry,^11^ a particularly useful feature of PDS is its ability to provide information about distance distributions.

Complementary to this, electron-nuclear double resonance (ENDOR) spectroscopy can be used to study the hyperfine (HF) interaction between nuclei and paramagnetic centers.^12–18^ This provides access to distances at and below the lower distance limit of PDS. Particularly, ^19^F ENDOR has been introduced recently as a method of choice to measure distances up to 15 Å.^19^ This approach is based on measuring the dipolar coupling between a nitroxide and a fluorine atom. The scarcity of ^19^F in biomolecules provides excellent selectivity and various labelling strategies exist.^20–22^ Furthermore, ^19^F ENDOR complements distance measurements based on nuclear magnetic resonance (NMR).^23,24^ The use of ^19^F ENDOR has been extended to various paramagnetic spin labels such as trityl radicals,^25,26^ Gd^3+^ ^27–29^ and Cu^2+^ ^30^ and methodological studies focused on ENDOR data analysis^31–33^ have been presented recently. Representative biological applications, such as studying the radical transfer process in ribonucleotide reductase^34^ and the solution state structural dynamics of a fluoride sensing riboswitch^35^ were reported. Also, the feasibility of ^19^F ENDOR on proteins in-cell was demonstrated.^36^

^19^F ENDOR spectra are mostly recorded as frequency domain experiments using the Mims ENDOR sequence.^37^ The resolution limit for small hyperfine couplings (corresponding to long distances) is determined by the splitting observed in the ^19^F ENDOR spectrum. Thus, understanding the origins of the ENDOR line broadening, which could mask this splitting, is crucial to extend the accessible range of measurements. Moreover, this is mandatory for a more rigorous extraction of distance distributions from ENDOR spectra. Approaches used in PDS, for example Tikhonov regularization, can in principle be adapted for ENDOR.^31^ Alternatively, a simulation of the ENDOR spectrum can be based on a model distance distribution, for example a simple Gaussian or a distribution derived from molecular modelling.^26,35,36,38^ However, the broadening mechanisms of the ENDOR spectrum, which are absent in PDS methods, give rise to ambiguity in the determination of distance distributions.

While internuclear couplings have enabled distance measurements in NMR,^39–41^ they have so far been mostly neglected in EPR and ENDOR spectroscopy, though recently their impact on the electron spin decoherence has been underlined.^42–45^ Here, we use the rational design of a rigid model system, frequency and time domain^46,47^ ENDOR experiments as well as spin dynamics simulations, using a formalism developed in-house,^48^ to disentangle unresolved interactions in ^19^F ENDOR spectra. So far, exclusively frequency domain ENDOR techniques were reported in conjunction with ^19^F ENDOR. Here, we present the first application of time domain Fourier transform ^19^F ENDOR. The importance of identifying the broadening mechanisms is demonstrated on a ^19^F and nitroxide-labelled RNA, where the intrinsic line width is predicted based on a structural model and spin dynamics simulations.

## 2 Results and discussion

### 2.1 Experimental design

**Fig. 1:**
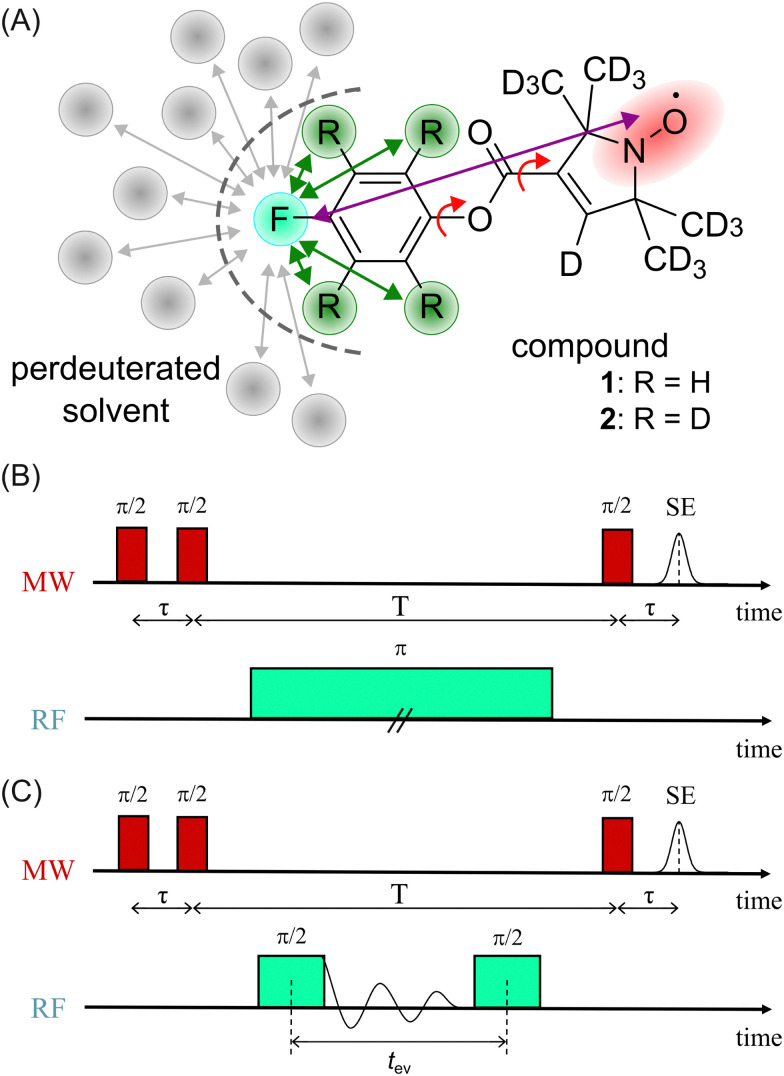
(A) Chemical structure of model compounds 1 and 2; red arrows indicate single bonds that allow rotational flexibility, purple arrow indicates the distance between the fluorine atom (turquoise) and the electron spin density (red), green arrows indicate intramolecular NDCs (relevant positions highlighted in green), gray arrows indicate potentially relevant intermolecular NDCs to the solvent; (B) Mims ENDOR sequence (frequency domain); (C) Mims-type time domain ENDOR sequence.

In a first step, we investigated the spectral broadening of ^19^F ENDOR spectra of a semi-rigid nitroxide-fluoride model system (Fig. 1(A)) with a point-dipole interspin distance of *r*(F–NO) ≈ 10.4 Å.^19^ In the previous study we showed that this simple system possesses reduced conformational flexibility and we assumed an ENDOR line width parameter of about 20 kHz.^19^ From this value, we estimated an accessible upper distance limit of 15–16 Å. However, we did not have a physical interpretation of this line width parameter except that it might be limited by the artificial power broadening of the pulse sequence, as explained below. To examine attainability of ENDOR line widths smaller than 20 kHz, a value which was generally the lower boundary reproduced in literature,^25–28,30,32,34,36^ we postulated that chemical shielding (CS) anisotropy^32^ and nuclear dipolar couplings (NDCs) should be considered. For this we designed the model system as well as the experimental approach such that these interactions can be modulated.

We chose an external magnetic field of 1.2 T where the influence of the CS anisotropy is attenuated and the concentration sensitivity is improved^26^ as compared to the previous study^19^ at 3.4 T. Overlap of ^1^H and ^19^F ENDOR resonances was circumvented by using deuterated pyrrolin-*N*-oxyl moieties, which we synthesized using established methods^19,49–52^ (Appendix S1, ESI†). To study the influence of NDCs, both intramolecular interactions with the nuclei of the phenyl ring and intermolecular interactions with the nuclei of the solvent were considered (Fig. 1(A)). The latter were reduced by using perdeuterated solvents (DMSO-*d*_6_ and CD_3_OD with v/v = 40/60). For the intramolecular NDCs it was possible to perform a H/D replacement reaction^53^ in the fluoroaryl substituent of 1 to obtain the fully deuterated analogue 2. Different NDCs of *D*_dip_(1) = 6.5 kHz and *D*_dip_(2) = 0.99 kHz are expected for the two vicinal protons/deuterons in both compounds. *D*_dip_ is defined as:^54^1
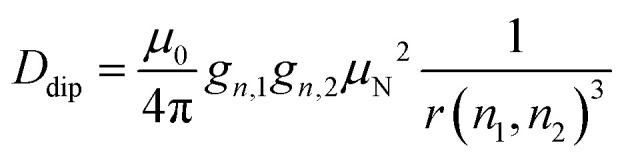
Here, *μ*_0_ is the vacuum permeability, *μ*_N_ the nuclear magneton, *g*_*n*,1/*n*,2_ the nuclear *g*-values^55^ and *r*(*n*_1_,*n*_2_) the internuclear distance.

For both model compounds, frequency and time domain (FD and TD) ENDOR experiments were performed. The former employs a single radio frequency (RF) π pulse to excite individual nuclear spin packages. The RF is varied on each point of the *x*-axis to record the spectrum stepwise. Here, the Mims-ENDOR pulse sequence^37^ was used for excitation and detection (Fig. 1(B)). In contrast, TD ENDOR experiments^46^ use π/2 RF pulses with constant frequency (here set to the ^19^F nuclear Larmor frequency) and aim to excite all nuclear transitions at once. The first π/2 RF pulse generates nuclear coherences. The nuclear signal is recorded in the time domain as a free induction decay (FID). After a certain evolution time *t*_ev_ a second π/2 RF pulse converts the nuclear coherences into nuclear spin polarisation. This affects the EPR echo intensity, which is recorded as a function of *t*_ev_. Here, we used a Mims-type TD ENDOR experiment (Fig. 1(C)). Importantly, the single RF pulse in FD ENDOR can cause broadening of the ENDOR line, so called power broadening,^56^ whereas TD ENDOR does not suffer from this effect.^57,58^

We note, that in both experiments the microwave (MW) pulses do not excite the whole EPR spectrum and therefore an orientation selection effect is observed. Due to the design of the model system (^19^F substituent in *p*-position of the phenyl ring) this effect is pronounced at the *B*_0_‖*g*_*z*_ observer position, even at 1.2 T. This is particularly beneficial for our study because the anisotropic contribution of the HF interaction to the ENDOR line width is strongly attenuated. Thus, we will only discuss ENDOR data obtained at *B*_0_‖*g*_*z*_, while the EPR spectrum and ENDOR data from other observer positions are provided in Appendix S2 and S3 (ESI†).

### 2.2 Frequency domain experiments and power broadening

**Fig. 2:**
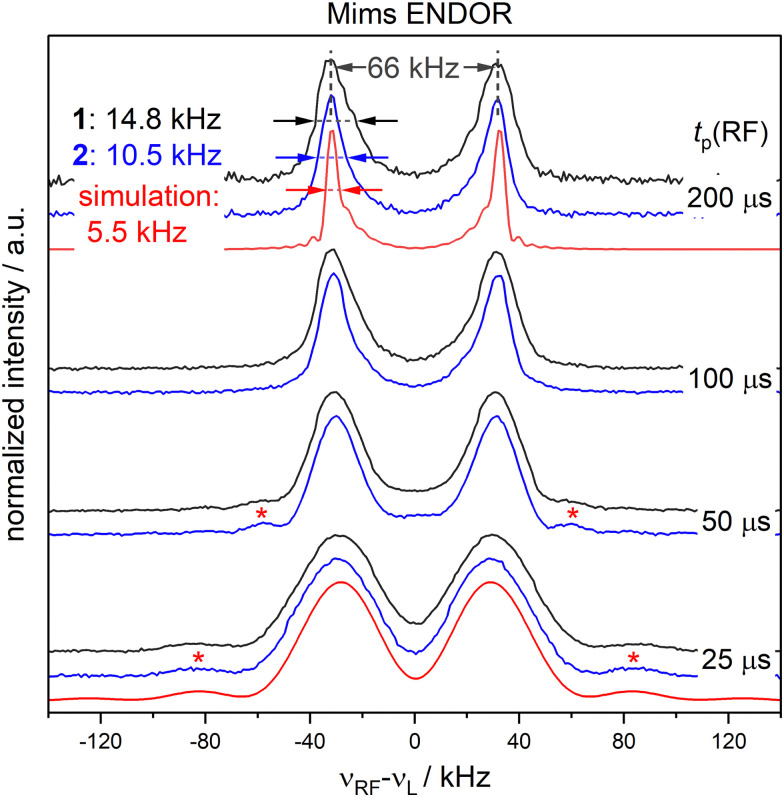
FD ^19^F Mims ENDOR experiments of 1 (black) and 2 (blue) with different RF pulse lengths; spin dynamics simulations (red) accounting for power broadening are shown for *t*_p_(RF) = 200 and 25 μs; red asterisks indicate positions where artifical peaks are induced by the RF pulse and arrows indicate the overall peak broadening (spectral broadening).

To examine the influence of power broadening due to the RF pulse in FD ENDOR experiments (Fig. 1(B)), spectra of 1 and 2 were recorded with different RF π pulse lengths. For each pulse length, the RF power was adjusted to obtain an optimal inversion according to the Rabi oscillations (see Materials and methods and Appendix S4, ESI†). Fig. 2 shows that the ENDOR lines are significantly broadened when using RF pulses with *t*_p_(RF) < 100 μs (see also Appendix S4, ESI†). Additionally, line shape alterations occur, which are particularly notable in the experiments with *t*_p_(RF) = 25 μs and 50 μs. There, peaks are artificially generated at both the low and high-frequency sides of the ENDOR spectrum, as indicated by asterisks in Fig. 2. These structures are not visible in the spectra measured with longer RF pulses (*t*_p_(RF) ≥ 100 μs). However, using long RF pulses reduces the sensitivity due to relaxation effects. As a main result we observe that peak broadening (Fig. 2, arrows) for 1 and 2 becomes significantly different using long pulses (14.8 ± 0.7 kHz *vs.* 10.5 ± 0.7 kHz, respectively). In contrast, almost no difference is observed with shorter pulses. This effect is confirmed by simulations (red lines, Fig. 2) as discussed in Section 2.4. Thus, using long RF pulses we were able to increase the resolution below 20 kHz and new, more subtle broadening mechanisms were unmasked. In the case at hand, NDC must be one of those, given that 1 shows a significantly larger line width than 2.

### 2.3 Time domain ENDOR experiments

**Fig. 3:**
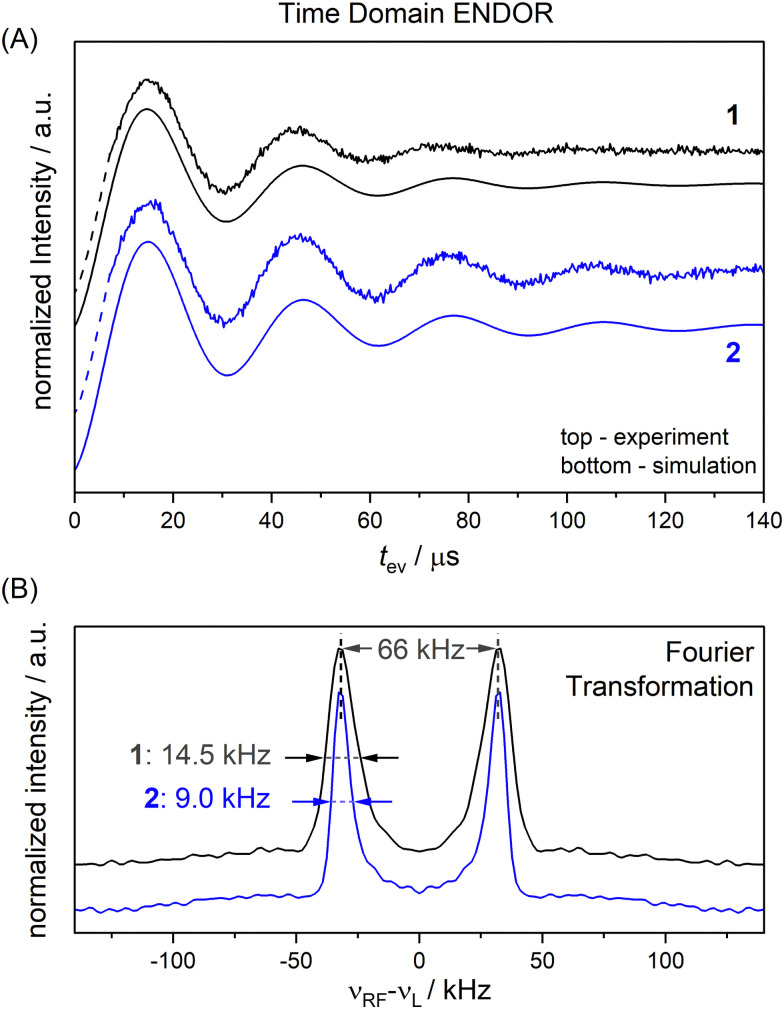
TD ENDOR experiments of 1 (black) and 2 (blue); (A) time traces (top) with simulations convoluted with a suitable exponential decay to match the experiment (bottom); the simulations were used to reconstruct the dead time of the experiment (dashed lines) to perform Fourier transformation; (B) Fourier transformed spectra of the time traces in panel A.

To completely remove the effect of power broadening we conducted TD ENDOR experiments (Fig. 3(A)). We observed a clear oscillation due to the fluorine FID for both compounds. The damping in the time trace is correlated to the spectral line width in the frequency domain. The modulation of the time trace of 2 lasts longer than of 1. This is in agreement with the trend in the line width (Fig. 2, top spectra) observed in FD ENDOR and confirms the NDCs to the neighbour protons as a major contribution to the line width for 1. To perform Fourier transformation of the time traces, spin dynamics simulations were used to reconstruct the dead time (Fig. 3(B)), for details see Appendix S5 (ESI†). The Fourier transformed (FT) spectra have full width at half-maximum (FWHM) values of 14.5 ± 0.2 kHz for 1 and 9.0 ± 0.2 kHz for 2. This is in good agreement with the line widths observed in the FD ENDOR spectra for 200 μs RF pulses and confirms that the residual width observed for soft RF pulses in FD ENDOR originates from sources other than power broadening.

**Fig. 4:**
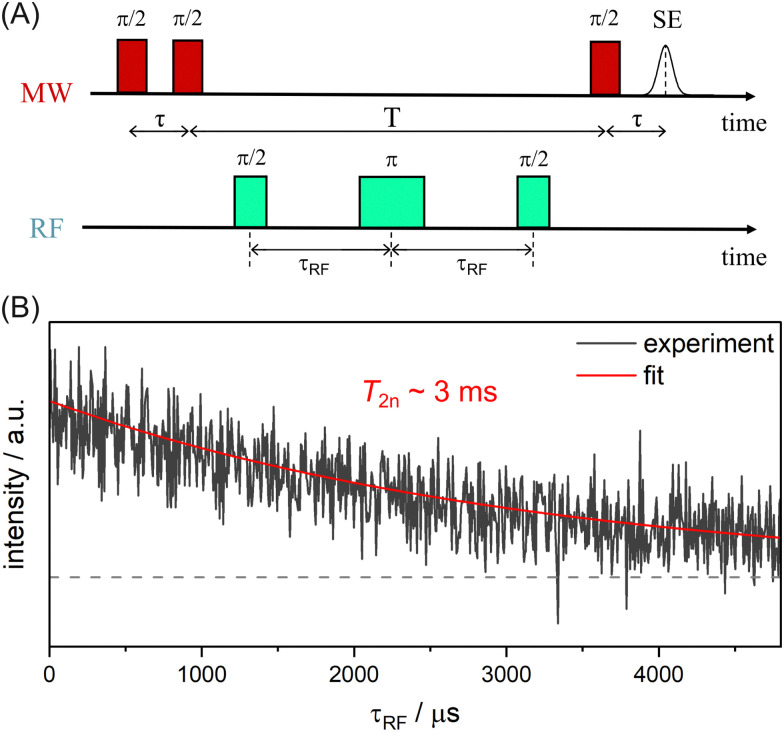
TD ENDOR experiment for measuring the *T*_2n_ relaxation time; (A) pulse sequence; (B) experimental data for 1 (black) with exponential fit (red), dashed line indicates zero intensity.

The nuclear relaxation time *T*_2n_ can be accessed *via* an extended TD ENDOR experiment.^46,59^ By inserting a refocusing RF π pulse into the sequence, a nuclear spin echo is created. *T*_2n_ can be measured by incrementing the time interval *τ*_RF_, thereby detecting the decay of the nuclear spin echo (Fig. 4(A)). We observe a nuclear *T*_2n_ relaxation time in the order of 3 ms for model compound 1 (Fig. 4(B)). This value is in good agreement with the estimate of *T*_2n_ ≈ 2·*T*_1e_ (with *T*_1e_ ≈ 1.5 ms), which is valid assuming that *T*_2n_ is dominated by the coupling to the fluctuating electron spin.^60^

### 2.4 Spin dynamics simulations

**Fig. 5:**
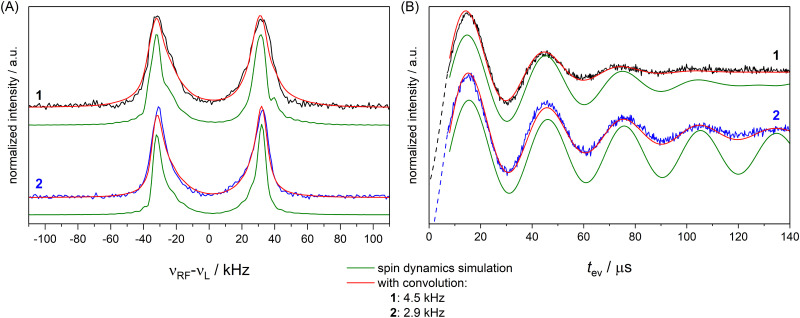
FD ENDOR spectra (A) with *t*_p_(RF) = 200 μs from Fig. 2 and TD traces (B) from Fig. 3 of 1 (black) and 2 (blue) with spin dynamics simulation (green); simulations convoluted with an additional Lorentzian line broadening of 4.5 kHz (1) and 2.9 kHz (2) shown in red.

To investigate the effect of the pulse sequence and the different magnetic interactions on the ^19^F ENDOR signal, we performed spin dynamics simulations. They are based on a numerical solution for the evolution of the spin density matrix during the ENDOR pulse sequences.^48^ Details are described in the Materials and methods section. Power broadening was intrinsically considered as effect of the pulses and we can explicitly include CS and NDC terms in the spin Hamiltonian as well as relaxation contributions. CS tensors for implementation in ENDOR simulations can be predicted by DFT calculations and, based on previous experiences,^32,33^ should represent reasonable approximations of the true physical values. Values for the NDCs can be calculated from eqn (1) using the internuclear distance extracted from an optimized DFT structure. Here, we consider only the two vicinal protons/deuterons, which have the largest NDC constants. All CS and NDCs and dipolar HF tensor values used for the simulation of compounds 1 and 2 are listed in Table 1. For the EPR parameters of the nitroxide spin system *g* = [2.00886, 2.00610, 2.00211], *A*(^14^N) = [15, 11, 95.8] MHz and *P*(^14^N) = [1.2, 0.5, −1.8] MHz were used from our previous work at 263 GHz^33^ and are in good agreement with the EPR spectrum here recorded at 34 GHz (Fig. S4, ESI†). We first simulated all FD experiments of 1 and 2 with and without NDCs (Fig. S7 in Appendix S4, ESI†). The simulations without NDCs show reasonable agreement with the experiment for *t*_p_(RF) < 100 μs, where power broadening dominates, whereas the simulations deviated from the experiment both in line width and shape for the spectra measured with longer RF pulses (red lines, Fig. 2). As expected, other contributions dominate the line shape. To investigate these other contributions, we focussed on TD ENDOR and the FD measurements with *t*_p_(RF) = 200 μs. We tested different combinations of CS, NDCs and *T*_2n_ effects. Simulations with *T*_2n_ effect also included an electronic *T*_2e_ relaxation term (eqn (6) and (9)), using the experimental *T*_M_ value of 1.5 μs, which however did not show any effect and is not discussed further. The simulations are shown in Fig. S12 and S13 in Appendix S6 (ESI†) and indicate that the NDC is a major contribution for the spectral broadening of 1 both for the TD and FD ENDOR experiments, while both the CS and nuclear transversal relaxation (in the order of magnitude >1 ms) are only minor contributions. Simulations (Fig. S18, ESI†) also indicate that the degree of deuteration for 2 being less than 100% did not significantly contribute to the broadening. *B*_0_ inhomogeneities and intermolecular NDCs can also contribute to broadening. We tested the former contribution by reducing the sample size (height) from 10 to 2 mm. The ENDOR spectra resulted identical (Fig. S16 in Appendix S7, ESI†). We also compared the spectra in perdeuterated solvent matrix with a 50% protonated solvent (Fig. S17, ESI†). We observe a broadening of the ENDOR peak by about 4 kHz, indicating that solvent NDCs also play a role. In our experiments, they were reduced by using deuterated solvents. Thus, especially in biological systems, the environment of the ^19^F atom may induce relevant NDCs and should be considered in detail, as illustrated in the next section. Spin dynamics simulations for both 1 and 2 are shown in Fig. 5. The CS and NDCs for a four spin system (a nitroxide radical, one fluorine atom coupled to two vicinal protons or deuterons) are included for both compounds. Relaxation effects are considered only for 1 due to high computational cost for 2. To reproduce the broadening of the FD ENDOR experiment and the damping of the TD ENDOR time trace, an additional convolution with a Lorentzian function or its time domain equivalent exponential decay function with a width of 4.5 kHz (1) or 2.9 kHz (2) was required. This could be due to some structural heterogeneity affecting all parameters of the spin Hamiltonian (HF coupling, CS and NDC). This residual broadening could be simulated by a small distance distribution with a FWHM of 0.5 Å (see Fig. S15, ESI†). Such small heterogeneities are expected also in rigid molecules, therefore it becomes difficult to distinguish them from other even more subtle mechanisms. We cannot exclude contributions of other relaxation pathways or the interaction with more distant nuclei as additional source of broadening. Indeed, the calculated system is still reduced to a four spin system (electron spin, ^19^F, two closest H/D) by neglecting contributions of other nuclear spins to the ENDOR spectrum, but this represents the current limit of our computational capabilities.

Simulation parameters for 1 and 2, CS values are taken from DFT calculations and NDCs calculated based on the molecular structure and eqn (1). As both the HF coupling and the NDC is assumed as dipolar the Euler angle *γ* is set to 0. The NDC decreases with increasing interspin distance and only values for the two closest atoms were included in the simulations. For the two other positions the NDCs amount to 1.1 kHz (H) and 0.17 kHz (D)

**Table:** 

	*T*_⊥_/kHz	*α*_HFC_/°	*β*_HFC_/°
1 and 2	66	161	1

**Table:** 

	*σ*_*xx*_/ppm	*σ*_*yy*_/ppm	*σ*_*zz*_/ppm	*α*_CS_/°	*β*_CS_/°	*γ*_CS_/°
1 and 2	205	310	360	29	14	−87

**Table:** 

	*D*_dip,1_/kHz	*α*_NDC,1_/°	*β*_NDC,1_/°	*D*_dip,2_/kHz	*α*_NDC,2_/°	*β*_NDC,2_/°
1	6.5	−17	143	6.5	−48	34
2	0.99	−17	143	0.99	−48	34

### 2.5 Application to an RNA duplex

**Fig. 6:**
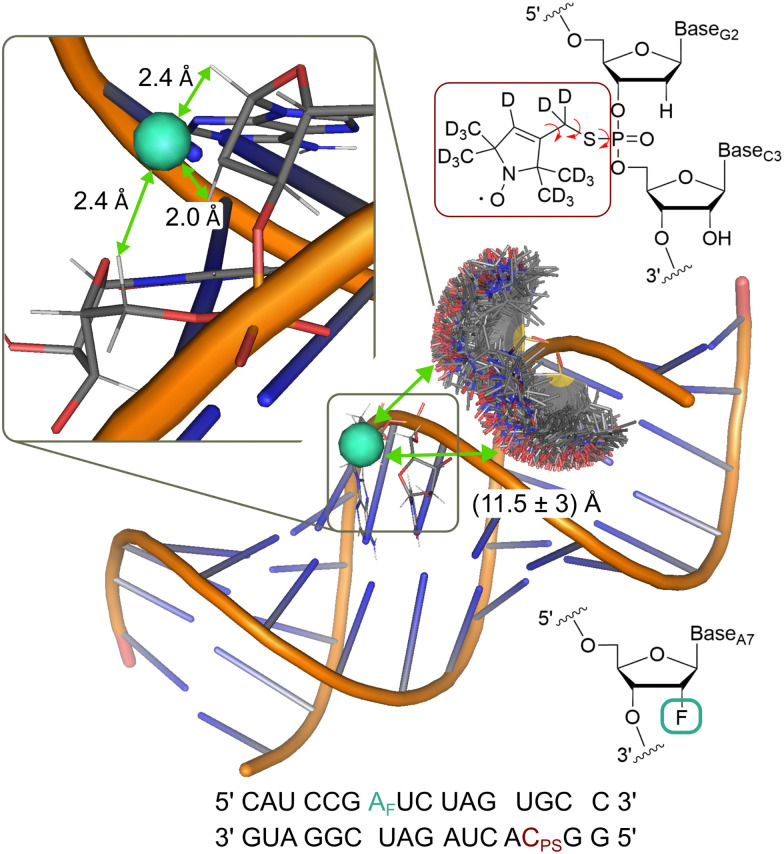
Model of the RNA construct with rotamer clouds predicted by MtsslSuite^62^ for the nitroxide spin label; indicated ^19^F-nitroxide distance is an average from the predicted distribution; inset shows enlarged region of the fluorine labelling position with the three closest protons, used for simulations; chemical structure of the nitroxide (red) and fluorine (turquoise) spin label and their position in the RNA backbone are shown in the top and bottom right, respectively.

**Fig. 7:**
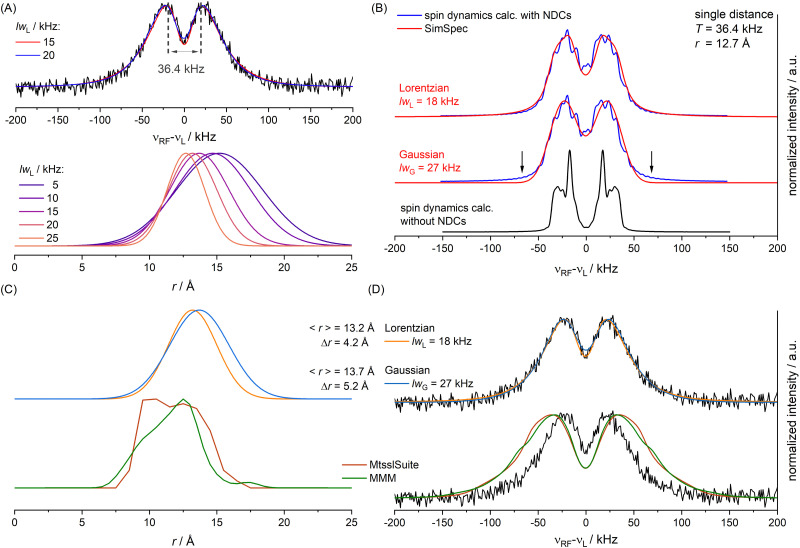
(A) For different *lw*_L_ parameters different unimodal Gaussian distributions (bottom) lead to optimal simulations of the ENDOR spectrum (top, black), simulations for *lw*_L_ = 15 and 20 kHz shown in color; *T* value used for spin dynamics calculation indicated in grey; (B) spin dynamics simulations including the NDCs (blue) in comparison to a calculation without NDCs (black) and SimSpec simulations (red) with optimized *lw* parameter for either Lorentzian or Gaussian convolution; (C), top: optimized Gaussian distribution for a Lorentzian *lw*_L_ parameter of 18 kHz (orange) or a Gaussian *lw*_G_ parameter of 27 kHz (blue); (C), bottom: distribution predicted by MtsslSuite (brown) or MMM (green); (D) simulation of the ENDOR spectrum for the optimized Gaussian distributions (top) or the predicted distributions (bottom) shown in panel C.

To demonstrate the analysis on a system with more conformational flexibility, we used an RNA construct with both a deuterated nitroxide spin label and a fluorinated nucleotide (Fig. 6). Due to the flexibility of the nitroxide and the presence of two diastereromers,^61^ the distance distribution is expected as a major source of broadening of the ENDOR spectrum. Frequency domain ENDOR experiments were performed at 34 GHz at four field positions in the EPR line (Fig. S19 in Appendix S8, ESI†). RF pulses with *t*_p_(RF) = 200 μs were used to reduce the effect of power broadening. We note that, in this case, TD ENDOR was not feasible with our commercial instrumental set-up due to the larger width of the ENDOR spectrum and the limited bandwidth of our RF pulses. For analysis of the FD experiments, the sum of spectra at the four field positions was used. The sum of the recorded experimental spectra is reported in Fig. 7(A).

To account for distance distributions in the simulations, a faster, frequency-domain type of static simulations with software such as SimSpec^33^ or EasySpin^63^ become indispensable due to computational cost. These approaches compute the resonance of a spin packet from a given Hamiltonian and require a spectral line width parameter (*lw*) with its corresponding line shape to account for unresolved broadening. To examine the effect of *lw* in determining a distance distribution with these approaches, we first simulated the ENDOR spectrum as a function of *lw*_L_ (FWHM of a Lorentzian) assuming a unimodal Gaussian distance distribution. For each distance of the distribution the four orientation selective spectra were computed using SimSpec and summed. Each calculated spectrum as a function of *r* was weighted with its probability and finally the spectra for all distances *r* in the distribution were summed up. This simulation approach was recently reported by Remmel *et al.*^35^ The residuals (root mean square deviations, RMSDs) for different *lw*_L_ parameters were examined (Fig. S22, ESI†) and showed that one can obtain simulations by different combinations of the *lw*_L_ parameter and the Gaussian distribution parameters (mean distance 〈*r*〉, FWHM Δ*r*). Specifically, we found that varying *lw*_L_ between 5 and 25 kHz, both the mean of the distribution (15.2 Å to 12.7 Å) and the FWHM (7.5 Å to 3.5 Å), can be adapted to reproduce the spectra (Fig. 7A and Fig. S21, ESI†) with best fits obtained for *lw*_L_ = 15 to 20 kHz. We note that from power broadening one would expect *lw*_L_ ∼ 5 kHz which does not result in a good fit of the spectrum (Fig. S21, ESI†). This illustrates the difficulty in determining a distance distribution if spectral line broadening is not known.

As outlined for the model compounds, possible broadening mechanisms and a suitable *lw* parameter can be identified by inspecting the environment of the fluorine nucleus. For this, we used a model of the RNA structure (see Materials and methods) and found three protons in close vicinity (Fig. 6). The NDCs of these three protons were calculated using eqn (1) and are listed in Table S3 (ESI†). The largest coupling is about 14 kHz and two others amount to 8 kHz. These NDCs are on the order of the *lw* parameters mentioned above and thus become relevant in the analysis.

A spin dynamics simulation was performed for a single distance (*T* = 36.4 kHz read from signal maxima, see Fig. 7(A)) including the NDCs. The simulation computed the powder pattern as a sum over four orientation selected spectra. Simulation parameters are given in Appendix S9, Table S3 (ESI†). This is shown in Fig. 7(B) with (blue line) and without NDCs (black line). CS anisotropy and *T*_2n_ effects were neglected as only minor effects were expected. Without NDCs (Fig. 7(B), bottom) we obtained the expected Pake pattern for the hyperfine dipolar interaction. It is evident that the NDCs completely alter the shape of the Pake pattern and cause substantial broadening (Fig. 7(B), blue trace). This raised the question how to approximate the combined effect of NDCs and power broadening in the faster simulation routines, if these effects are not included. To examine this, we approximated these broadening effects in SimSpec simulations, by using either a Lorentzian or Gaussian *lw* parameter (Fig. S23, ESI†). A Lorentzian shape with a *lw*_L_ = 18 kHz best reproduced the spectrum (Fig. 7(B), top, red trace), consistent with the values found initially (*lw*_L_ parameters between 15 and 20 kHz). As a comparison, the Gaussian fit also reproduces the spin dynamics simulation (*lw*_G_ = 27 kHz) except for the flanks of the spectrum (Fig. 7(B), arrows). Note also the difference in the *lw* parameter value, arising from the different forms and definitions of the Gaussian *vs.* Lorentzian functions. Although, according to the RMSDs (Fig. S23, ESI†), the Lorentzian shape is preferred, we performed the subsequent analysis with distance distributions for both line shapes (*lw*_L_ = 18 kHz and *lw*_G_ = 27 kHz).

The static (frequency domain) simulations of the experimental spectrum using distance distributions were repeated based on the obtained *lw* values, which were now kept fixed. Again, the two parameters of the Gaussian distribution (〈*r*〉 and Δ*r*) were varied systematically and the RMSD was evaluated. For *lw*_L_ = 18 kHz we found a minimal RMSD for 〈*r*〉 = 13.2 Å and Δ*r* = 4.2 Å. For *lw*_G_ = 27 kHz we found a minimal RMSD for 〈*r*〉 = 13.7 Å and Δ*r* = 5.2 Å (Fig. 7(C), top). Considering the similar quality of the best simulations (Fig. 7(D), top) the results suggest uncertainties introduced by the line shape parameter are on the order of ≳0.5 Å (〈*r*〉) and ≳1 Å (Δ*r*).

Finally, we compared the obtained distributions with predictions of the spatial orientation of the spin label using MtsslSuite^62^ and MMM.^64^ Overall, the predictions turn out in reasonable agreement with the obtained Gaussian distributions (Fig. 7(C), bottom), but suggest a higher abundance of shorter distances. This deviation may be correlated to the achievable accuracy of about 2–4 Å for the used modelling tools as reported previously.^65^ As a control, simulations of ENDOR spectra using the modelled distributions (Fig. 7(D), bottom) are not consistent with the experimental spectrum due to overestimation of short distances (≲10 Å) in the conformer modelling.

## 3 Conclusion

We have demonstrated that spectral line broadening in a frequency domain ENDOR spectrum is determined not only by power broadening, which can be strongly attenuated, but in particular by nuclear dipolar couplings. We observe a strong H/D isotope effect in the spectrum of a fluorinated nitroxide model system, reaching an unprecedented ENDOR line width of ∼9 kHz. This narrow width in principle should allow to resolve dipolar splittings as low as ∼9 kHz, corresponding to distances up to ∼20 Å with nitroxides. We note that recently Gd^3+^ (*S* = 7/2) has been proposed to extend the accessible distance range up to 20 Å.^28^ Our result strongly suggests that by removing the line broadening effects, especially NDCs and power broadening, the accessible distance range could be generally extended. The results also showed that the ENDOR line width is not limited by the electronic phase memory time (*T*_M_), which was here ∼1.5 μs. Also, we could measure *T*_2n_ ∼ 3 ms using time domain ENDOR and found that *T*_2n_ in this order of magnitude has a negligible effect on the line width. However, the time domain fluorine FID signals of the model compounds had to be convoluted with an additional line width of ∼3–4 kHz, the origin of which we could not assign. At the low magnetic field used here (1.2 T) the CS anisotropy can be neglected but it may become relevant at higher magnetic fields, which will have to be investigated case-by-case.

We showed, that knowledge of the spectral broadening mechanisms is important for analysis of inter-spin distances in biomolecules, where conformational distributions become a major contribution to the ENDOR spectrum. The broadening obtained from spin dynamic simulations under consideration of NDCs was consistent with the range of values obtained by fitting of *lw* and a unimodal Gaussian distance distribution using FD static simulations. However, we show that the *lw* parameter can be constrained in advance, setting an important boundary in the analysis. NDCs can be estimated from molecular modelling and, in absence of spin dynamics simulations, considered directly in a static simulation. This becomes potentially critical for evaluation of more complex distance distributions. Interestingly, the range of *lw* parameters reported so far in the literature extend from about 10 to 50 kHz (Table S4, ESI†), suggesting that, in many cases, the *lw* parameters have been correlated with the fitted distance distribution. Finally, the approach presented in this study can be readily transferred to other paramagnetic (radicals or metal ions)-^19^F systems to estimate spectral line widths of individual distances. Thus, this should provide a starting point for more rigorous analysis of distance distributions from ^19^F ENDOR spectra, for instance using Tikhonov regularization^31,66^ or Bayesian methods.^33,67^

## 4 Material and methods

### 4.1 Synthesis and sample preparation

Synthesis of the deuterated nitroxide model systems was based on established procedures.^19,49–53^ Some additional information on the final synthesis steps of 1 and 2 along with analytics is presented in Appendix S1 (ESI†). If not indicated otherwise, the samples were prepared as solutions with 500 μM concentration in deuterated DMSO-*d*_6_ and CD_3_OD (v/v = 40/60). DMSO (Eurisotop) and methanol (Sigma-Aldrich) both had an initial deuteration degree of 99.8% according to the manufacturers. Since the solvent bottles had been opened before, we controlled the proton content by NMR and estimated a proton content of about 1% for both solvents. The radical concentration was chosen relatively high to achieve sufficient S/N ratios for the performed analysis. A volume of about 10 μL was filled into quartz capillaries (1.6 mm OD, Wilmad 222T-RB) and frozen in liquid nitrogen.

For the RNA labelling, RNA strands with an internal fluorine modification at the 2′ position of the A7 sugar in strand A and a phosphorothioate modification at C3 in strand B (Integrated DNA Technologies) were purchased freeze dried and dissolved to a concentration of 2 mM in D_2_O and 500 μM in H_2_O, respectively. The spin labelling of strand B was performed according to the protocol from Qin *et al.*,^61^ details given in Appendix S8 (ESI†).

The complementary RNA strands were combined in a 1 : 1 ratio with 1× PBS in D_2_O. The mixture was heated to 95 °C for 2 minutes and slowly cooled down to 60 °C within 30 minutes and further to room temperature within 15 minutes. 33% glycerol-*d*_8_ were added as cryoprotectant. The RNA duplex concentration was adjusted to 300 μM in the final sample. 12 μL of the sample were transferred into a DEPC treated quartz capillary (1.6 mm OD, Wilmad 222T-RB) and flash frozen in liquid nitrogen.

### 4.2 34 GHz EPR and ENDOR spectroscopy

EPR and ENDOR measurements were performed at 50 K using a Bruker E580 X/Q-band spectrometer equipped with a Bruker EN 5107D2 pulse EPR/ENDOR resonator in a CF935 helium gas flow cryostat (Oxford Instruments). A 170 W TWT amplifier (Model 187Ka, Applied Systems Engineering) and a 600 W RF amplifier (600A225A Amplifier Research) were used to amplify MW and RF pulses, respectively. The EPR spectrum of 1 along with ENDOR observer positions is reported in Fig. S4 in Appendix S2 (ESI†). Electronic relaxation data are very similar to previously reported ones at 3.4 T,^19^ measurements are shown in Fig. S5 (ESI†). The EPR spectrum of the RNA sample is shown in Fig. S19 (ESI†) along with observer positions used, measurements of *T*_1e_ and *T*_M_ for the delay used for ENDOR experiments are shown in Fig. S20 (ESI†).

For FD Mims ENDOR measurements (π/2–*τ*–π/2–π_RF_–π/2–*τ*-echo), MW pulse lengths of 12 ns (π/2) and RF pulse lengths of 25 to 200 μs (π) were used. The combinations of RF power and pulse lengths were determined using nuclear transient nutation experiments (Appendix S4, ESI†) at the *B*_0_‖*g*_*z*_ observer position. For 1 and 2 the delay time *τ* was set to 2000 ns based on previous measurements^19^ and shot repetition times (SRT) corresponding to five times *T*_1e_ were used, corresponding to 10.5 and 8.8 ms for 1 and 2, respectively. For the RNA sample *τ* was set to 2500 ns and SRT = 15 ms was used, corresponding to about five times *T*_1e_. The RF sweep was performed with stochastic acquisition mode with one shot per point (SPP). The echo integration was performed in a 24 ns window placed symmetrically around the maximum of the echo intensity.

For TD Mims ENDOR measurements (π/2–*τ*–π/2–π/2_RF_–t–π/2_RF_–π/2–*τ*-echo) MW pulse lengths of 12 ns (π/2) and RF pulse lengths (π/2) of 6 μs were used. The frequency of the RF pulses was set to the ^19^F nuclear Larmor frequency (approx. 48.1 MHz). The delay time *τ* was optimized and set to 1600 ns. A delay of 200 μs between the last RF pulse and MW pulse was needed to avoid spectral artefacts. The second RF pulse was moved in 240 ns steps. A 4-step RF phase cycle [0,0] − [0,π] − [π,0] + [π,π] was used as reported earlier.^57^ The echo integration was performed in a 70 ns window for 1 and 90 ns for 2 placed symmetrically around the maximum of the echo. All TD ENDOR experiments were recorded with 10 SPP and SRT of 8 ms. Details of the Fourier transformation procedure are provided in the Appendix.

For the nuclear relaxation measurement (π/2–*τ*–π/2–π/2_RF_–*τ*_RF_–π_RF_–*τ*_RF_–π/2_RF_–π/2–*τ*-echo) model compound 1 was dissolved in a mixture of DMSO-*d*_6_ and glycerol-*d*_8_ (2 : 3), due to the favourable electron transversal relaxation time. The measurement was performed at the *B*_0_||*g*_*y*_ position of the EPR spectrum. The delay time *τ*_RF_ was increased in 6 μs steps and an 8-step RF phase cycle [0,0,0] − [0,0,π] − [π,0,0] + [π,0,π] + [0,π,0] − [0,π,π] − [π,π,0] + [π,π,π] was used, based on the proposed RF phase cycle for the TD Mims ENDOR measurement. All remaining time delays and pulse lengths were used in the same way as for the TD Mims ENDOR measurements. The echo integration was performed with a 160 ns window placed symmetrically around the maximum of the echo. The experiment was recorded with 10 SPP and SRT of 12 ms.

### 4.3 Spin dynamics simulations

Using spin Hamiltonians, relaxation superoperators and the Liouville–von-Neumann equation it is possible to calculate the evolution of the spin density matrix for an ENDOR experiment.^48^ First, this requires defining the spin Hamiltonians. For a nitroxide-fluoride spin system, two HF couplings with the electron spin need to be considered: the strong interaction with the ^14^N in the nitroxide ring and the interaction with ^19^F. As described in our previous publications,^32,33^ the EPR resonances can be calculated separately from the ENDOR resonances leading to the following two Hamiltonians:2
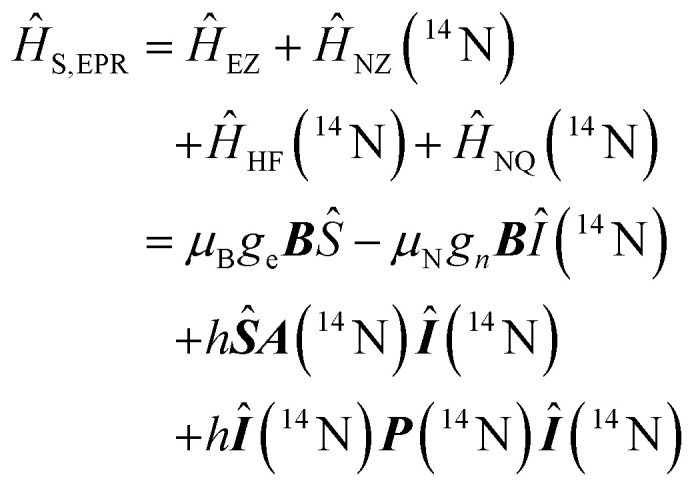
3
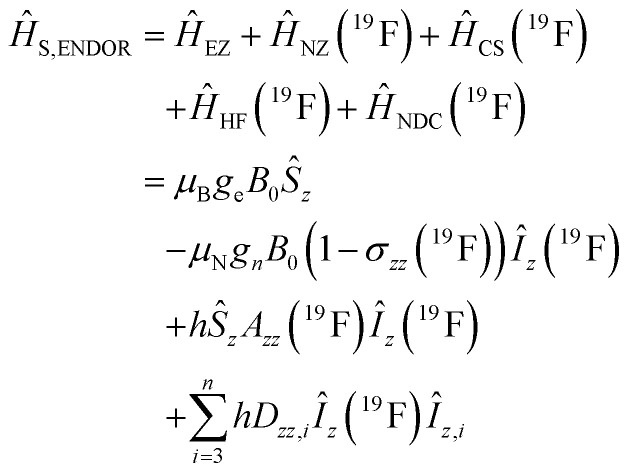
where ***B*** = [0,0,*B*_0_] and *h* = 6.62607015 × 10^−34^ J s is the Planck constant. The Hamiltonians consist of terms for the electron Zeeman interaction (EZ), the nuclear Zeeman interaction (NZ) and the HF coupling. For *Ĥ*_S,EPR_ the nuclear quadrupole (NQ) interaction is considered and for *Ĥ*_S,ENDOR_ the CS and NDC are included. The calculation is performed in angular frequency units 
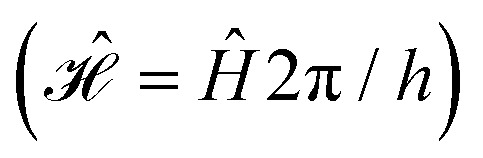
 and in an interaction frame, that accounts for the applied frequencies. This introduces offset terms Δ*ω*_S_ = *ω*_S_ − *ω*_MW_ and Δ*ω*_I_ = *ω*_I_ − *ω*_RF_ in dependence of the electron resonance frequency *ω*_S_, the nuclear Larmor frequency *ω*_I_ and the applied frequencies for the pulses *ω*_MW_ and *ω*_RF_. When pulses are applied, additional terms are considered for the MW pulse (*ω*_1e_*Ŝ*_*x*_) and the RF pulse (*ω*_2n_*Î*_*y*_).

In the first step of the calculation *Ĥ*_S,EPR_ is diagonalized and the EPR resonance frequency is determined for a set grid of orientations with respect to the magnetic field. Subsequently, a weighting factor for each orienation is determined according to the MW resonance and the excitation profile of the MW pulse.^32,33,68,69^ Then, spin dynamics simulations of the ENDOR spectrum are performed for each excited orientation. For this, *Ĥ*_S,ENDOR_ is used.

The initial spin density matrix *ρ̂*_0_ (for the chosen conditions 
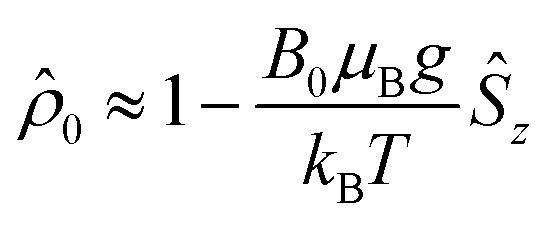
) can be propagated stepwise till the end of the pulse sequence using the solution of the Liouville–von-Neumann equation for time independent Hamilton operators:^70^4
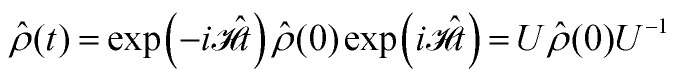


The signal intensity is then calculated as the expectation value of the *Ŝ*_*y*_ operator:

5 *I*_signal_ ∝ 〈*Ŝ*_*y*_〉 = Tr(*ρ̂*(*t*)*Ŝ*_*y*_)

The calculation of *ρ̂*(*t*) is repeated for every RF-value of the *x*-axis of the experiment. The calculated ENDOR spectrum represents a single orientation as it could be detected for a single crystal sample.^48^ All calculated spectra are weighed according to the excitation function and summed to represent the final spectrum.

Spin dynamics simulations can optionally include relaxation effects based on experimental parameters.^70,71^ Longitudinal relaxation is neglected in the analysis. The transversal relaxation is approximated to an exponential decay of coherences to zero in dependence of a rate constant *R*_2_, expressed by the relaxation time *T*_2_ (*R*_2_ = 1/*T*_2_).^70^ For a *S* = 1/2, *I* = 1/2 spin system, relaxation of electron (*R*_2e_), nuclear (*R*_2n_), double-quantum (*R*_2dq_) and zero-quantum (*R*_2zq_) coherence occurs. The following matrix expresses which elements of the spin density matrix in the {|*αα*〉, |*αβ*〉, |*βα*〉, |*ββ*〉} basis in Hilbert space are affected by transversal relaxation rates:6
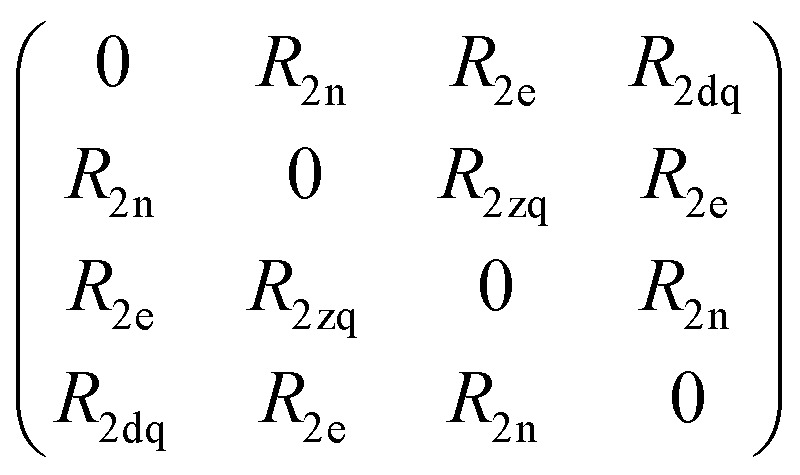
Here, only *R*_2e_ and *R*_2n_ were considered as *R*_2zq_ and *R*_2dq_ did not show any effect and no experimental value was available.^72^

The influence of relaxation on the spin density matrix is described in Liouville space instead of Hilbert space. There, the density matrix is transformed to a vector form: when the spin Hamilton operator *Ĥ*_S_ has the dimension *n* in Hilbert space, in Liouville space a superoperator 
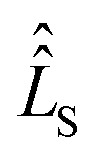
 of the dimension *n*^2^ is used:7
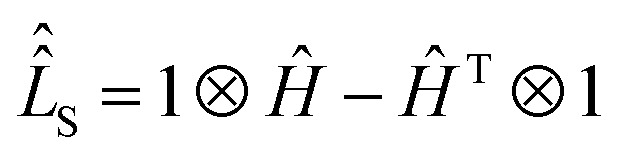
The time evolution under the time-independent Hamiltonian 
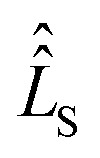
 with a relaxation superoperator 
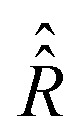
 is then given by:^70^8
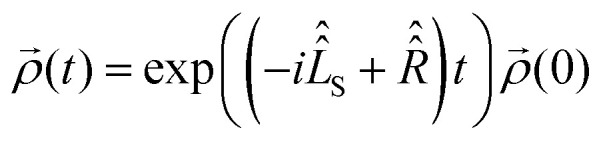
The relaxation superoperator induces changes in the matrix elements affected by the corresponding relaxation rate (here *R*_2e_ and *R*_2n_). Eqn (9) is exemplary for the decay of the |*αα*〉〈*αβ*| element:^70^9
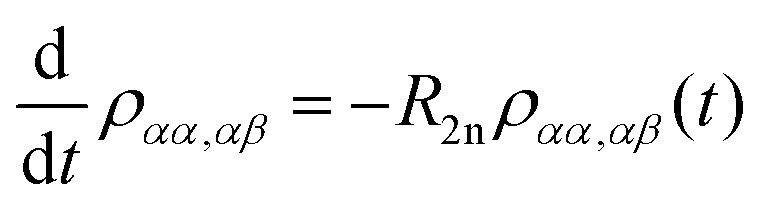
Further details on spin dynamics simulations and their implementation are provided in ref. 72. Compared to static simulations, spin dynamics simulations have a longer computational time (minutes *vs.* hours/days time scale). This is due to the repeated calculation of matrix exponentials for every point of the *x*-axis and for every excited orientation. The computational demand increases with the size of the spin Hamiltonian. Therefore, simulations including more NDCs or relaxation effects may lead to impractical runtimes.

### 4.4 DFT calculations and RNA modelling

DFT calculations were performed for 1 and 2. Based on recommendations by the Grimme group,^73^ the DFT geometry optimization was performed with Orca 5.0.3^74^ using the ωB97X-D4 functional with tight SCF convergence criteria, the def2-QZVP^75^ basis set, the auxiliary basis set def2/J,^76^ atom-pairwise dispersion correction based on tight binding partial charges (D4)^77,78^ and the RIJCOSX approximation.^79,80^ For the NMR parameter determination Orca 4.2.1^81^ was used with the PBE0 functional,^82,83^ the def2-TZVPP basis sets^75^ with the auxiliary basis set def2/JK^76^ and RIJK approximation.^84,85^

Modeling of the 16mer A-RNA helix was performed using the w3DNA server^86^ using an A-form helix with a twist and rise of 32.7° and 2.81 Å, respectively.^87^ The introduction of phosphorothioate modifications to the RNA backbone leads to the presence of two diastereomers. Both of them need to be considered when calculating possible rotamers of the spin label. This is already implemented in MMM. In MtsslSuite the diastereomeric labels have to be selected separately. The labels used were R5-TP in MMM and ‘bebRNA1’ and ‘bebRNA1diast’ in MtsslSuite. Additionally, the distance distributions from the rotamers to the fluorine were calculated.

## Author contributions

Conceptualization (AK, MB, AM); data curation (AK); data analysis (AK, LS, LR, MB, AM); funding acquisition (MB); investigation (AK, LS, LR, MLR, AM); methodology (AK, LS, LR, MB, AM); resources (MB); software (AK); visualization (AK, AM); manuscript writing (AK, MB, AM); discussion and review (all authors).

## Data availability

Experimental data and simulation scripts to perform the presented simulations are available on the open database Göttingen Research Online: https://doi.org/10.25625/AGW0Y9.

## Conflicts of interest

There are no conflicts to declare.

## Supplementary Material

CP-027-D4CP04443F-s001
