# Supplementary material for: Frequency and time domain 19F ENDOR spectroscopy: role of nuclear dipolar couplings to determine distance distributions
Source: Phys Chem Chem Phys. 2024 Dec 12;27(3):1415–25. doi: 10.1039/d4cp04443f (PMC11656155; doi:10.1039/d4cp04443f)
Supplement: CP-027-D4CP04443F-s001 [file CP-027-D4CP04443F-s001.pdf]

Supplementary Information (SI) for Physical Chemistry Chemical Physics.
This journal is © the Owner Societies 2024

Frequency and Time Domain ^{19}F ENDOR Spectroscopy: Role of Nuclear Dipolar Couplings to Determine Distance Distributions

Annemarie Kehl,[a] Lucca Sielaff,[a,b] Laura Remmel,[a] Maya L. Rämisch,[a,b] Marina Bennati[a,b] and Andreas Meyer[a,b]

E-mail: annemarie.kehl@mpinat.mpg.de, marina.bennati@mpinat.mpg.de, andreas.meyer@mpinat.mpg.de

[a]Research Group ESR Spectroscopy, Max Planck Institute for Multidisciplinary Sciences,
Am Fassberg 11, 37077 Göttingen, Germany.

[b]Georg-August-Universität Göttingen, Institute of Physical Chemistry,
Tammannstr. 6, 37077 Göttingen, Germany.

Contents

1 **Synthesis of Model Compounds** **S3**

2 **Echo-detected EPR spectra and relaxation measurements for model compounds** **S5**

3 **Orientation selective ENDOR at different observer positions** **S7**

4 **FD ENDOR with different RF pulses** **S8**

5 **TD ENDOR** **S11**

6 **Additional simulations of FD and TD ENDOR experiments** **S12**

7 **Additional line broadening contributions for the model compounds** **S17**

8 **Spin labelling of the RNA and spectroscopic characterization** **S20**

9 **Simulations for the RNA Construct** **S22**

10 **Line width parameters reported in literature** **S25**

11 **Analytics of deuterated *p*-fluorophenol, compounds 1 and 2** **S26**

1 Synthesis of Model Compounds

Pt/C (10 wt-%) and dicyclohexylcarbodiimid (DCC) were purchased from Sigma-Aldrich. Dimethylaminopyridine (DMAP) and 4-fluorophenol were purchased from Acros organics. Solvents and all remaining chemicals were purchased from either Sigma-Aldrich, Acros organics or TCI chemicals. All chemicals were used as received without further purification. Purification steps after syntheses were performed according to the references cited below. Analytical data are shown in section 11 of this appendix.

Deuterated 2,2,5,5-tetramethyl-3-pyrrolin-*N*-oxyl-3-carboxylic acid

The synthesis of the deuterated nitroxide building block is summarized in Figure S1. We closely followed procedures presented previously [1–4]. The descriptions in literature (see citations) give more detailed information on the synthesis procedure that was followed here. Our results mostly agree with what has been reported.

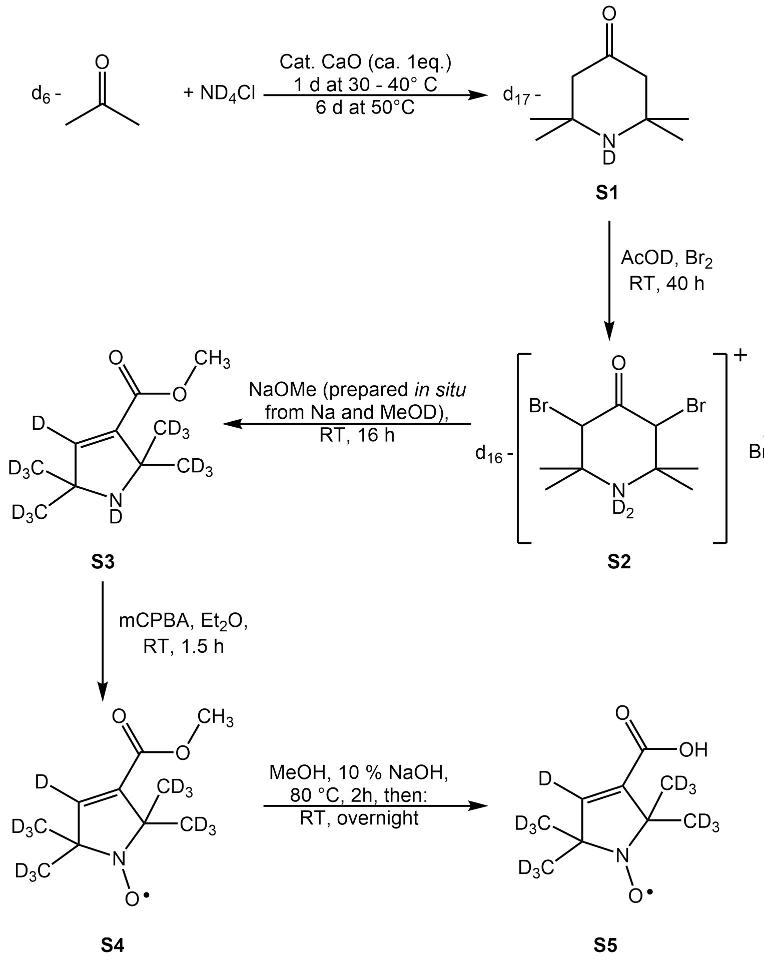

Figure S1: Synthesis of deuterated nitroxide.

Deuterated *p*-fluorophenol

Deuterated *p*-fluorophenol was prepared on a 120 mg scale as reported by Sawama *et al.* (Figure S2) [5]. Deuteration degrees of 92 and 91 % are estimated for H2 and H3, respectively, using an NMR-based method proposed by Sawama *et al.* (not shown). This value is comparable to what was reported by the same authors. As a side product of the H/D exchange, perdeuterated phenol is formed. This impurity amounts to about 15 % (see Appendix 11) and is difficult to separate [5].

EI-MS: *m/z* 116.1 (100 %, $[C_6D_4FOH]^+$, $[M]^+$).

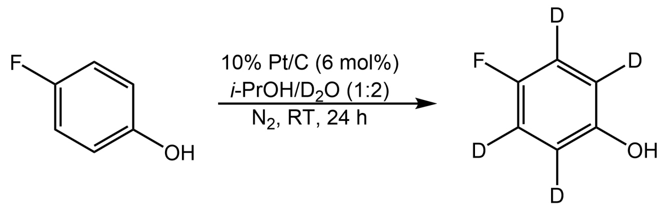

Figure S2: Synthesis of deuterated *p*-fluorophenol.

Steglich esterifications to obtain 1 and 2

1 and **2** were obtained by Steglich esterifications (Figure S3) as reported previously [6]. The purity of the compounds was assessed via liquid chromatography coupled with mass spectrometry (LC/MS, see SI 11). For **1**, a single peak was observed in the elugram. For **2**, two peaks were observed with relative intensities of ca. 1 : 5. Based on the respective mass spectra, the larger peak was assigned to **2** while the smaller of the peaks could be assigned to perdeuterated phenol. This is formed as a side product when performing the H/D exchange to obtain deuterated *p*-fluorophenol [5]. This side product is difficult to separate but does not affect our ^{19}F ENDOR measurements aside from slightly reducing the ENDOR efficiency.

ESI-MS for **1** : *m/z* 291.3 (60 %, $[C_{15}D_{13}H_4NO_3F]^+$, $[M]^+$).
ESI-MS for **2**: *m/z* 295.3 (60 %, $[C_{15}D_{17}NO_3F^+$, $[M]^+$).

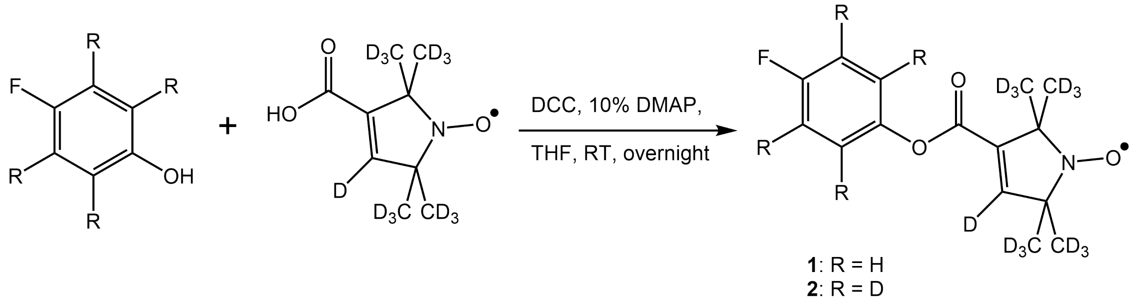

Figure S3: Steglich esterifications to prepare **1** and **2**.

2 Echo-detected EPR spectra and relaxation measurements for model compounds

Figure S4 shows the echo detected EPR spectrum of **1** along with a SimSpec simulation. The arrows indicate the observer positions used for ^{19}F ENDOR measurements shown in Figure S6. In the main text, only the $B_0 \parallel g_z$ is discussed, because almost single crystal-like ENDOR spectra are obtained at this position. The EPR spectrum of **2** is indistinguishable from that of **1**.

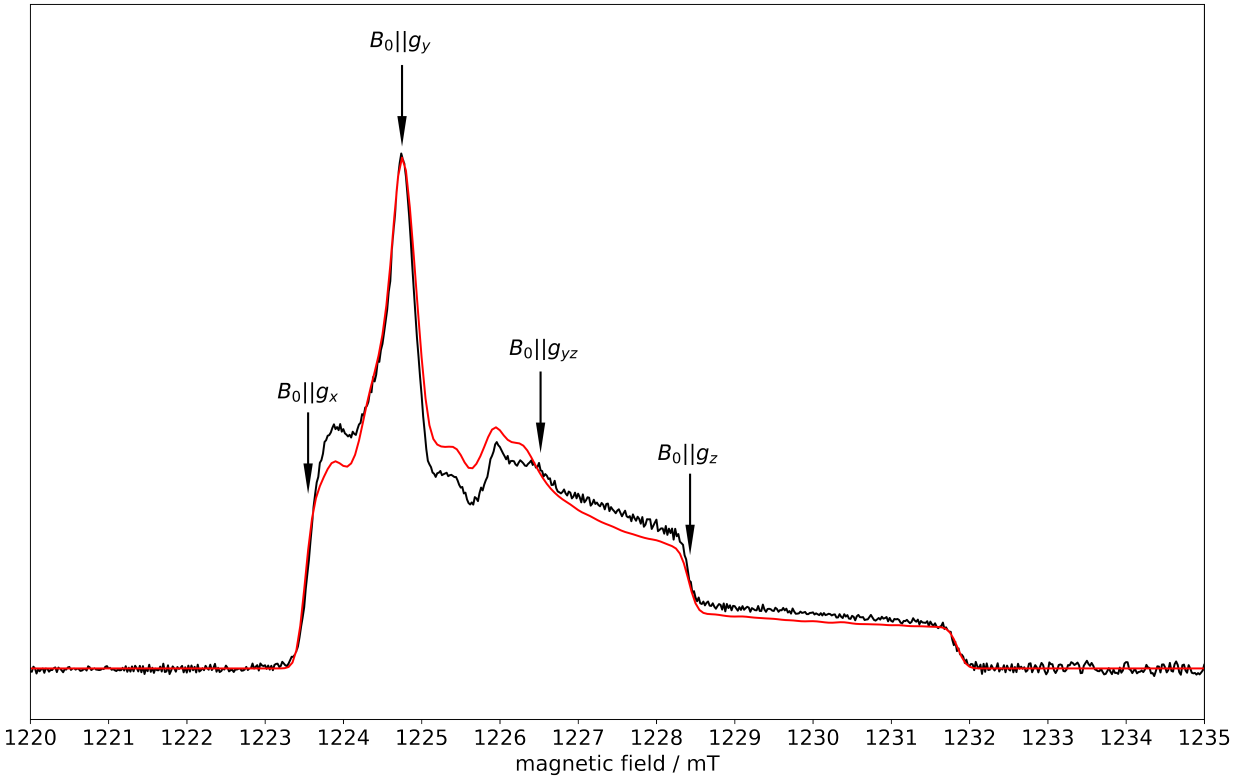

Figure S4: Echo detected EPR spectrum of **1** (black) and SimSpec simulation (red). Simulation parameters: g_x=2.00886, g_y=2.00610, and g_z=2.00211, $A_x(^{14}\mathrm{N})$=15 MHz, $A_y(^{14}\mathrm{N})$=11 MHz, and $A_z(^{14}\mathrm{N})$=95.8 MHz. The arrows indicate ENDOR observer positions (Figure S6 in SI 3).

Figure S5 shows T_{M} values that were measured in dependence of the delay time T_{RF} and the experimental data for $T_{\mathrm{RF}} = 50$ and 200 µs for both **1** and **2**.

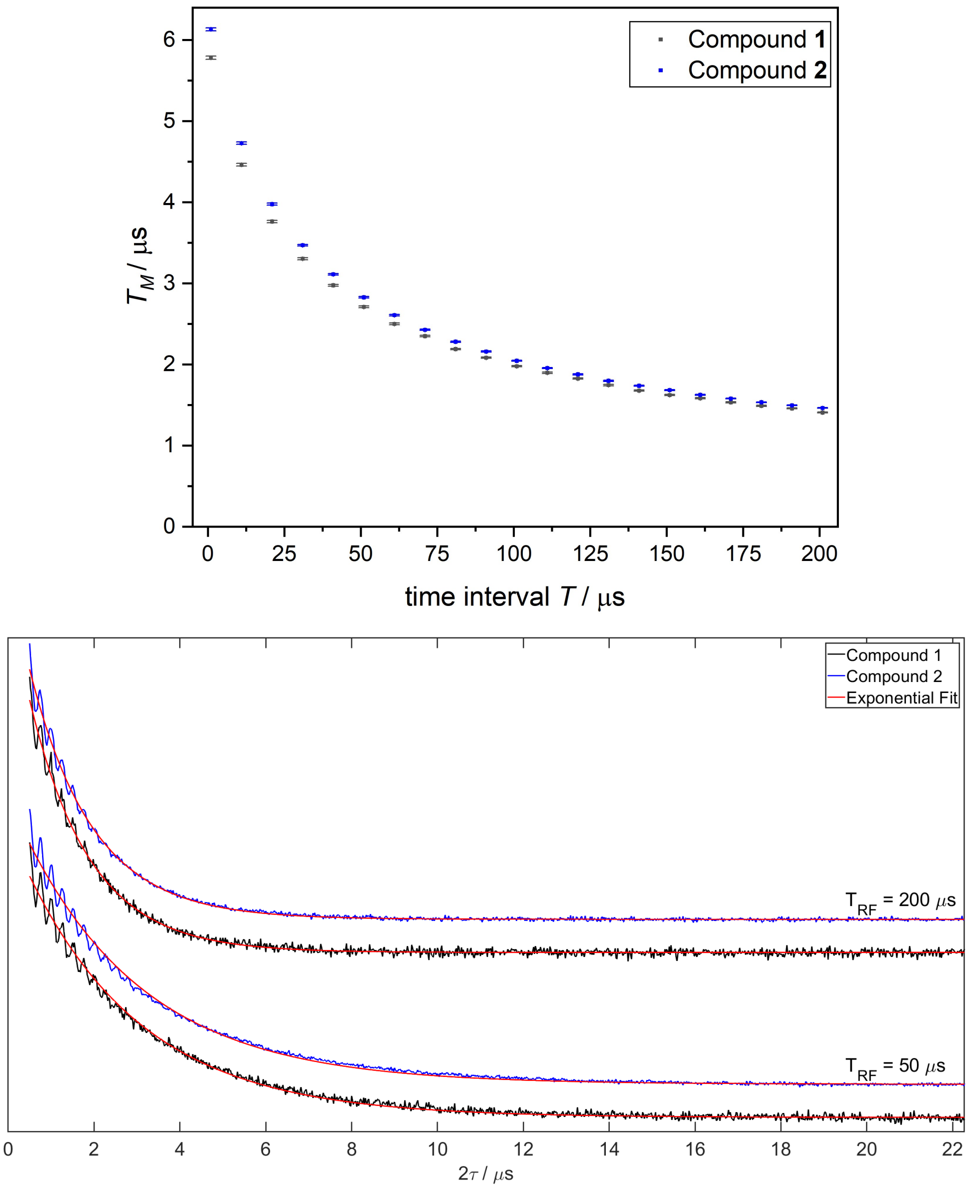

Figure S5: T_{M} values measured in dependence of T_{RF} (top) and experimental data for $T_{\mathrm{RF}} = 50$ and 200 µs (bottom) for **1** and **2**.

3 Orientation selective ENDOR at different observer positions

Figure S6 shows orientation selective ENDOR measurements of **2** performed with 200 µs RF pulses along with the sum spectrum of the orientation selective data, which shows typical Pake pattern like shape. These data were measured on a 300 µM sample, for which we did not observe any differences compared to our measurement results obtained on the 500 µM samples shown in the main text. For **1**, similar data were obtained with slightly broader lines at all observer positions (not shown).

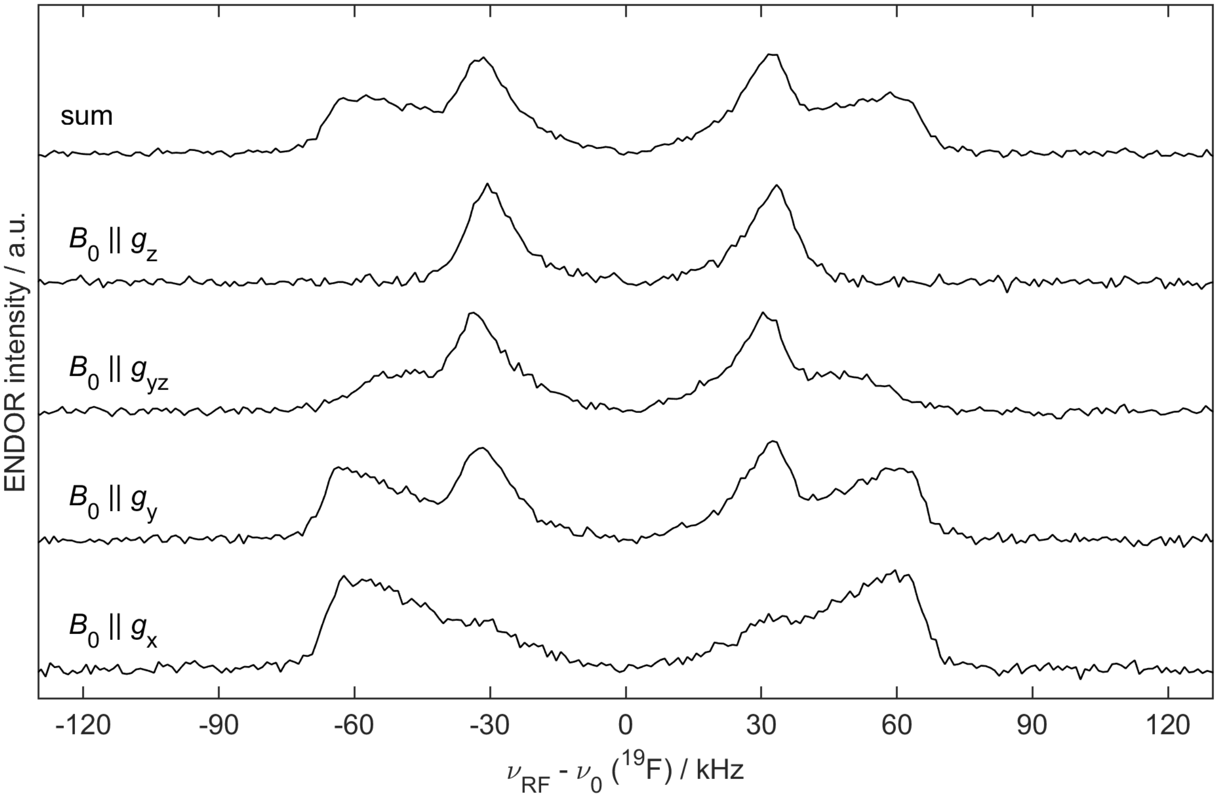

Figure S6: Orientation selective ENDOR measurements of **2** with 200 µs RF pulses (300 µM in a mixture of DMSO$-d_6$ and CD_3OD (1:1.5)).

4 FD ENDOR with different RF pulses

In Figure S7 all FD ENDOR experiments with different RF pulse lengths are shown. In panel A the spin dynamics simulations including the effect of power broadening (PB) are included, while in panel B the NDC is included as well. In panels C and D the FWHM values of all FD ENDOR experiments and simulations are shown. For the experiments uncertainties are determined using the procedure described below. The FWHM values are listed in Table S1 in Appendix 6.

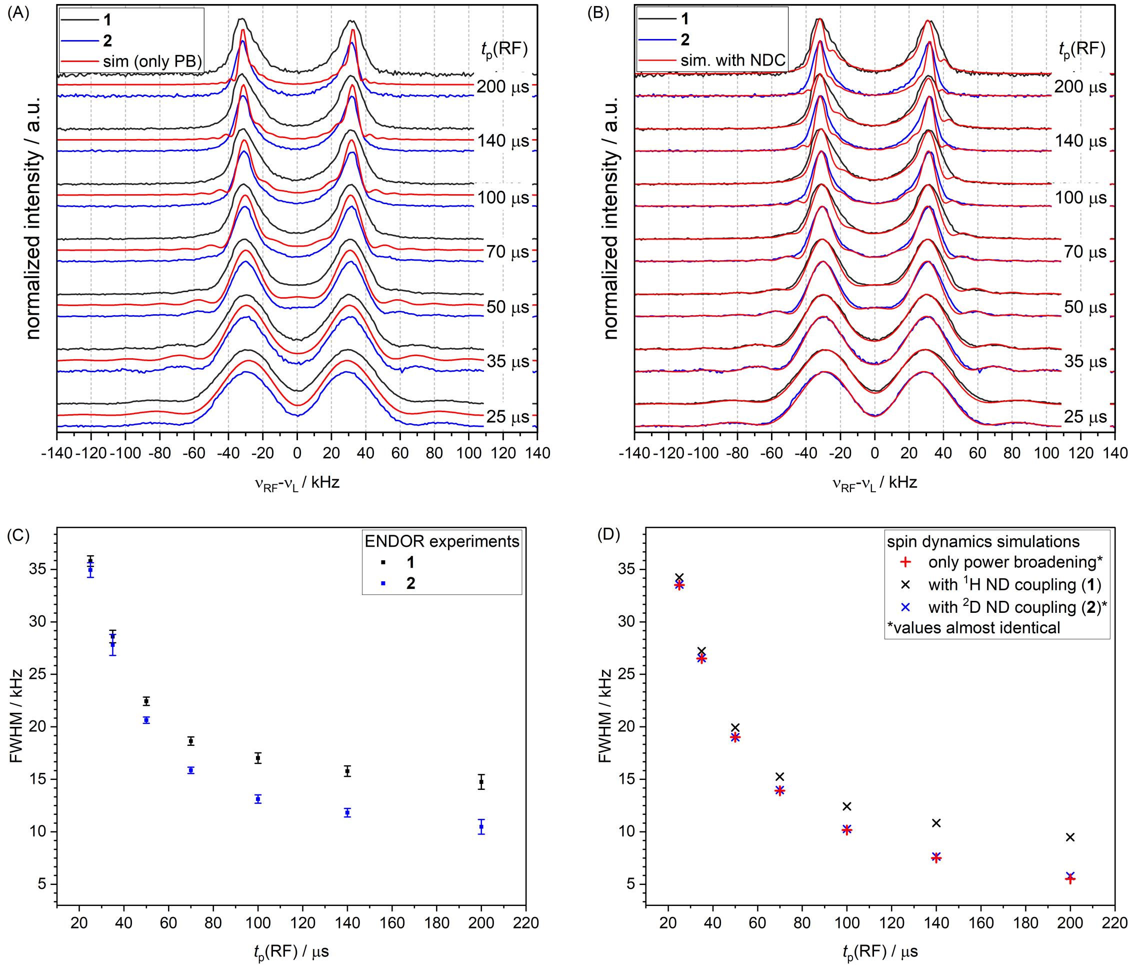

Figure S7: The effect of different RF pulse lengths in FD ENDOR. A and B: Mims ENDOR experiments for **1** (black) and **2** (blue) with spin dynamics simulations (red) including only PB (A) and additionally the NDC (B). C and D: FWHM values of the experiments with uncertainties of **1** (black) and **2** (blue) (C) and the simulations without NDC (red) and with NDC (black and blue, respectively) (D).

To give uncertainties for the FWHM of the experiments, an estimation of uncertainty is needed for the experiment. As described in ref. [7], an estimation of the noise is possible using a filtered version of the experiment if no statistical model is available for the data. As shown in Figure S8 (A) for the $t_{\mathrm{p(RF)}} = 50\,\mu\mathrm{s}$ experiment of **1** a Savitzky-Golay filter was used to generate a smooth spectrum. The difference between experiment and smoothed spectrum is considered as an approximation of the noise. Then, the standard deviation of the noise σ is calculated. In Figure S8 (B) the experiment is shown with areas of 95% confidence given by the $I(\exp) \pm 1.96\sigma$. For the three lines (experiment, upper and lower boundary) the intersection with 0.5 signal intensity is is determined as the FWHM and area of uncertainty.

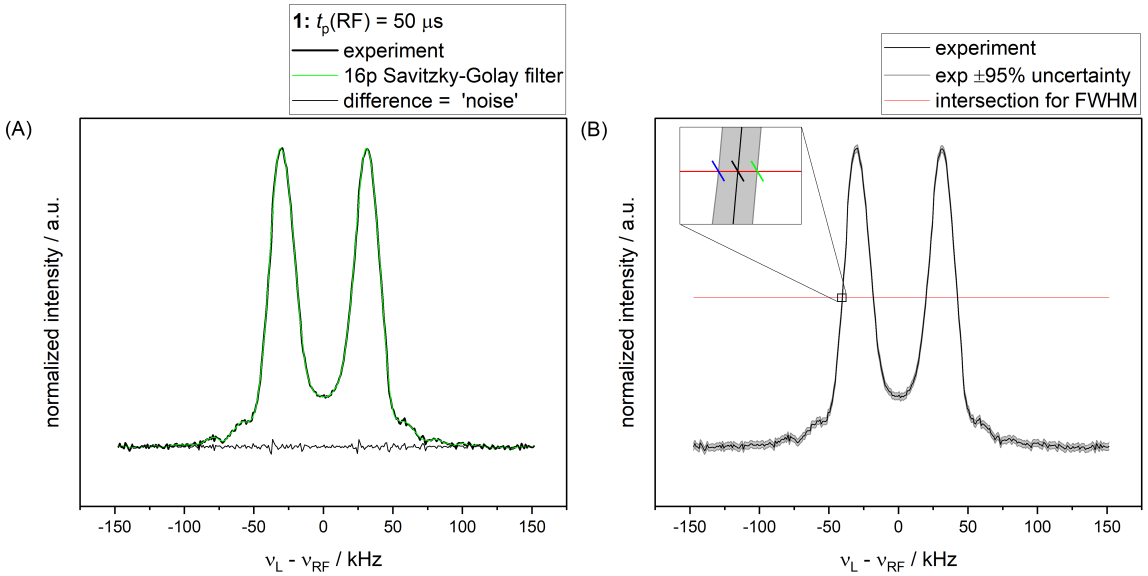

Figure S8: A: The difference between an experiment and a smoothed spectrum is considered as its noise; B: a confidence interval of 95%, i.e. 1.96σ, is added to the experiment to determine the FWHM with 95% uncertainty interval at the intersection with 0.5 intensity as shown in the inset.

To be able to adapt the RF pulse power for each RF pulse length nuclear nutation experiments were performed on **1** and **2** using a 600 W RF amplifier at 50% output power setting and with 8 dB RF attenuation. The results are shown in Figure S9. The first minimum occurs to a very good approximation at an RF pulse length of 50 μs in both cases. These measurements were also taken as point of reference for all other ENDOR measurements, in which we varied the RF power in steps of 3 dB, which corresponds to incrementing ω_{RF} and the RF pulse lengths by factors of $\sqrt{2}$ (see equations (S.1) and (S.2)).

$$\omega_{\mathrm{RF}} \propto \sqrt{P_{\mathrm{RF}}} \tag{S.1}$$

$$P_{\mathrm{RF}}(\mathrm{RF}-\mathrm{Attenuation}) = P_0 \cdot 10^{-\frac{\mathrm{RF}-\mathrm{Attenuation}}{10\mathrm{dB}}} \tag{S.2}$$

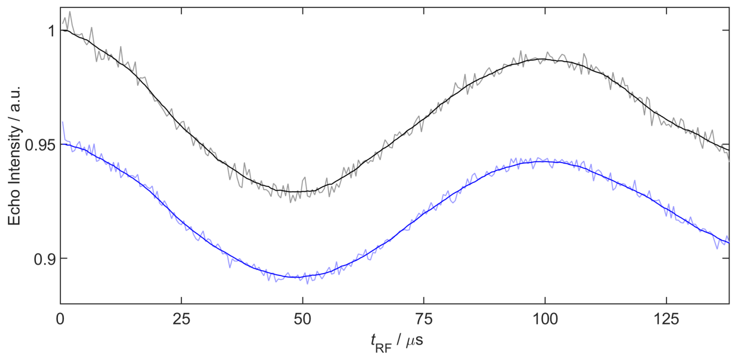

Figure S9: Nuclear nutation time traces of **1** (black) and **2** (blue) measured with a 600 W RF amplifier at 50 % output power setting and 8 dB attenuation. The position of the first minimum was taken as optimal pulse length. Semi-transparent lines are the original data, solid lines are the data after ten-point data smoothing (equal weighting) as implemented in Xepr.

5 TD ENDOR

To compare the line widths of the frequency and time domain ENDOR experiments, Fourier transformations of the obtained time traces were performed. The recorded time traces suffer from dead-time issues, due to the finite RF pulse length, which prevents a detection of a signal with $t_{\mathrm{ev}} = 0$. To avoid distortions in the Fourier transformation, it is necessary to reconstruct missing data points of the two time traces. Spin dynamics simulations with infinitely short RF pulses were performed yielding a dead-time-free simulation of the time traces. Based on the simulation the missing data points of the experimental time trace were reconstructed. The time traces were zero-filled to three times the length of the original data and Fourier transformed yielding the resulting spectra shown in the main text in Figure 3. The uncertainties of the line widths were calculated as described in Appendix 4. Note that no apodization has been performed since it introduces additional line broadening [8]. However, the missing apodization leads to baseline ripples in the Fourier transformation.

In Figure S10 the TD ENDOR experiments are shown together with the spin dynamics simulations including the NDC of either two protons (for **1**) or two deuterons (for **2**).

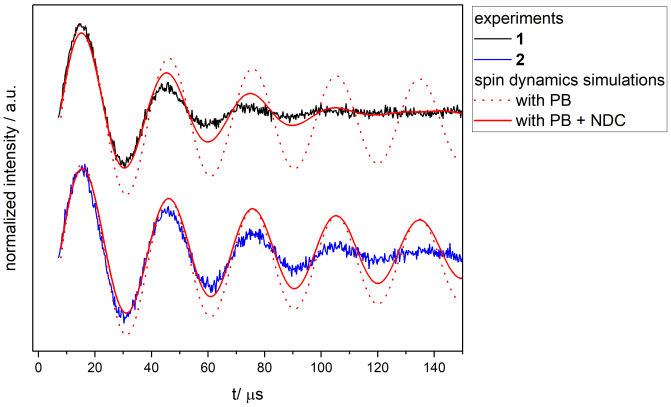

Figure S10: TD ENDOR experiments of **1** (black) and **2** (blue) with spin dynamics simulations with NDC (red line) and without NDC (dotted red line).

6 Additional simulations of FD and TD ENDOR experiments

Table S1 gives all FWHM values of the simulations and experiments with different RF pulse lengths for **1** and **2**.

Table S1: FWHM of ^{19}F ENDOR experiments and simulations of both compounds.

$t_p(\mathrm{RF})$ / µs	25	35	50	70	100	140	200
	FWHM / kHz						
exp (**1**)	35.8 ± 0.5	28.6 ± 0.6	22.4 ± 0.4	18.6 ± 0.4	17.0 ± 0.5	15.8 ± 0.5	14.8 ± 0.7
exp (**2**)	34.9 ± 0.7	28 ± 1	20.6 ± 0.3	15.9 ± 0.3	13.1 ± 0.4	11.8 ± 0.4	10.5 ± 0.7
sim. PB	33.5	26.5	19.0	13.9	10.2	7.5	5.5
sim. H NDC	34.2	27.2	19.9	15.3	12.4	10.8	9.5
sim. D NDC	33.6	26.6	19.0	14.0	10.3	7.7	5.8

In Figure S11 the FWHMs of the FD and TD ENDOR experiments ($t_{\mathrm{RF}} = 200$ µs for FD ENDOR) are plotted and compared with the values of the spin dynamics simulations with different effects for FD ENDOR. The exact values are given in Table S2. In Figure S12 and S13 simulations with all possible combinations of line broadening effects are plotted for both FD and TD ENDOR, respectively.

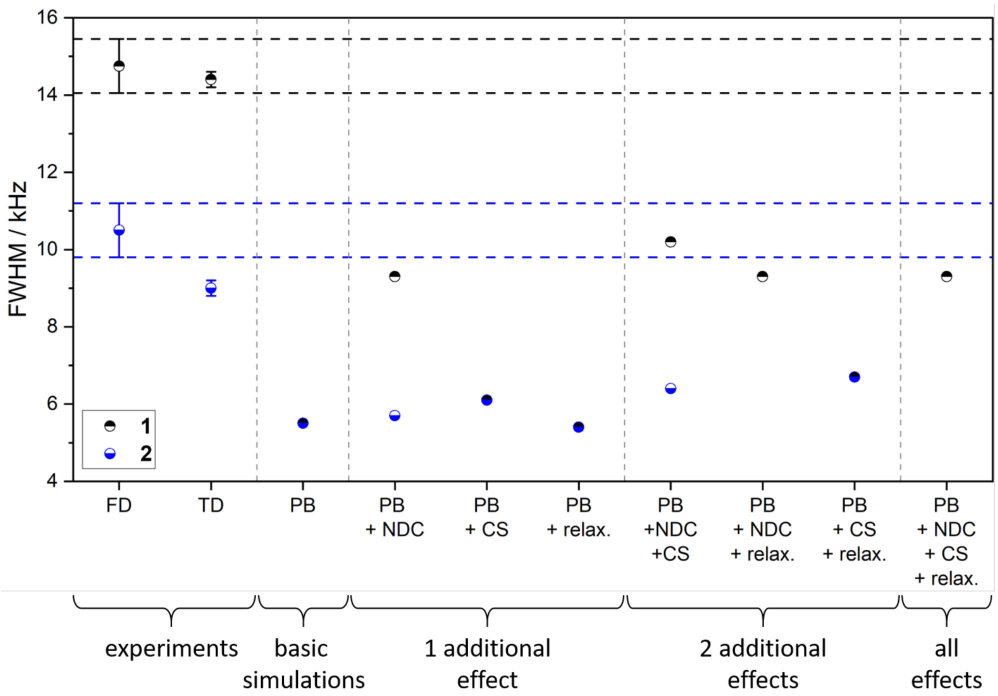

Figure S11: Summary of the FWHMs of the FD and TD ENDOR experiments with uncertainties ($t_{\mathrm{RF}} = 200$ µs for FD ENDOR) as well as FWHM values of the FD ENDOR spin dynamics simulations, dashed horizontal lines indicate areas of uncertainty for the FD ENDOR experimental values.

Table S2: FWHM values for all data points of the ENDOR experiments and simulations shown in Figure S11, included are FD and TD ENDOR experiments, power broadening, the NDC and CS terms as well as transversal nuclear relaxation with T_{2n} = 3 ms.

	**1**	**2**
**experiments**		
frequency domain	14.8±0.7	10.5±0.7
time domain	14.4±0.2	9.0±0.2
**spin dynamics simulations**		
PB	5.5	
PB + NDC	9.3	5.7
PB + CS	6.1	
PB + relaxation	5.4	
PB + NDC + CS	10.2	6.4
PB + NDC + relaxation	9.3	(n.d.)
PB + CS + relaxation	6.7	
PB + NDC + CS + relaxation	9.3	(n.d.)

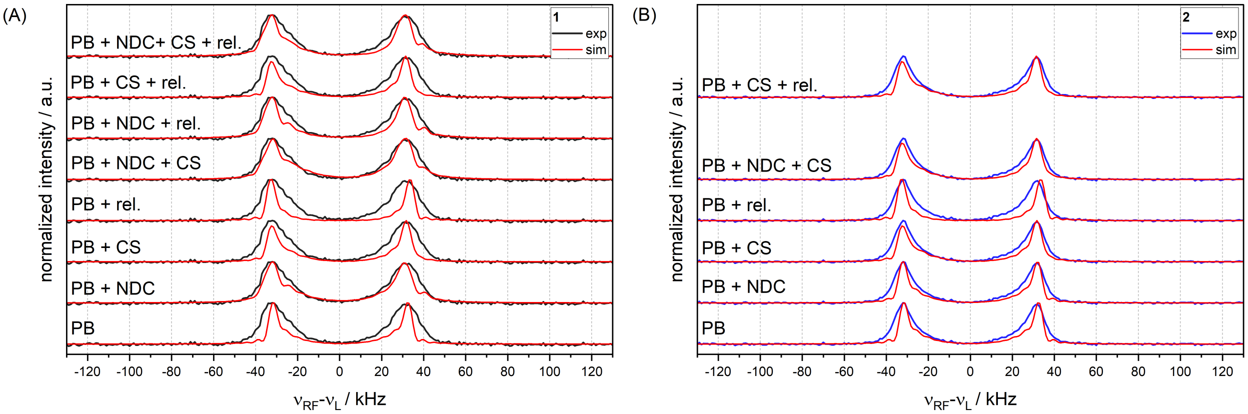

Figure S12: Simulations corresponding to all data points included in Figure S11, included are FD ENDOR experiments for comparison, data are shown for **1** (A) and **2** (B).

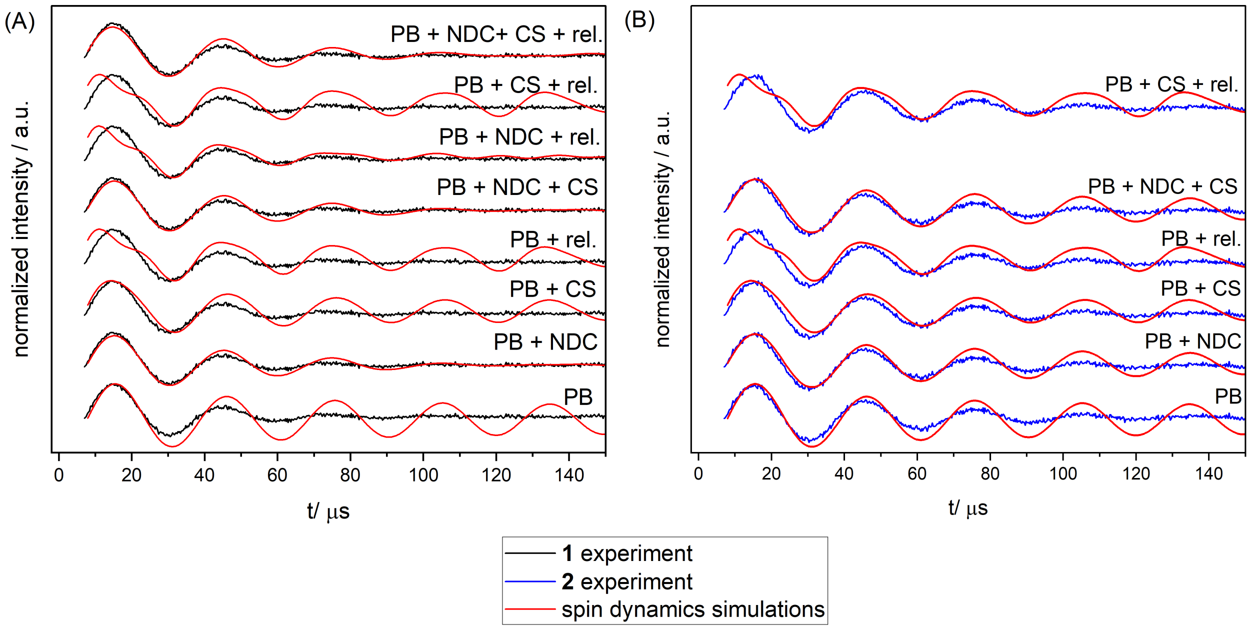

Figure S13: Simulations of TD ENDOR traces for all possible combinations of line broadening effects for **1** (A) and **2** (B). Similarly to the FD ENDOR simulations the label PB here indicates the simulation where no other effects are included, even though no actual PB effects are present in TD ENDOR.

In Figure S14 spin dynamics simulations (A) and their FWHM values (B) for different T_{2n} relaxation times including the CS and NDC (colored lines) in comparison to the experiment of **1** with $t_p(\mathrm{RF}) = 200\,\mu\mathrm{s}$ are shown. For the simulation in the main text $T_{2n} = 3\,\mathrm{ms}$ was used in accordance with the TD ENDOR relaxation measurement. The simulations show that any value for T_{2n} larger than $\sim 1\,\mathrm{ms}$ would not strongly impact the simulated line width.

A broadening contribution of structural heterogeneity to the ENDOR spectra of **1** and **2** can be simulated based on an intrinsic line width estimated from the spin dynamics simulations as shown for the RNA construct in the main text. This is shown in Figure S15. For this a Lorentzian lw_L parameter was estimated from the spin dynamics simulations (panel A) and the spectra simulated with different unimodal Gaussian distributions (panels B and C). A FWHM of the distribution (Δr) of 0.5 Å produced a good fit for both compounds.

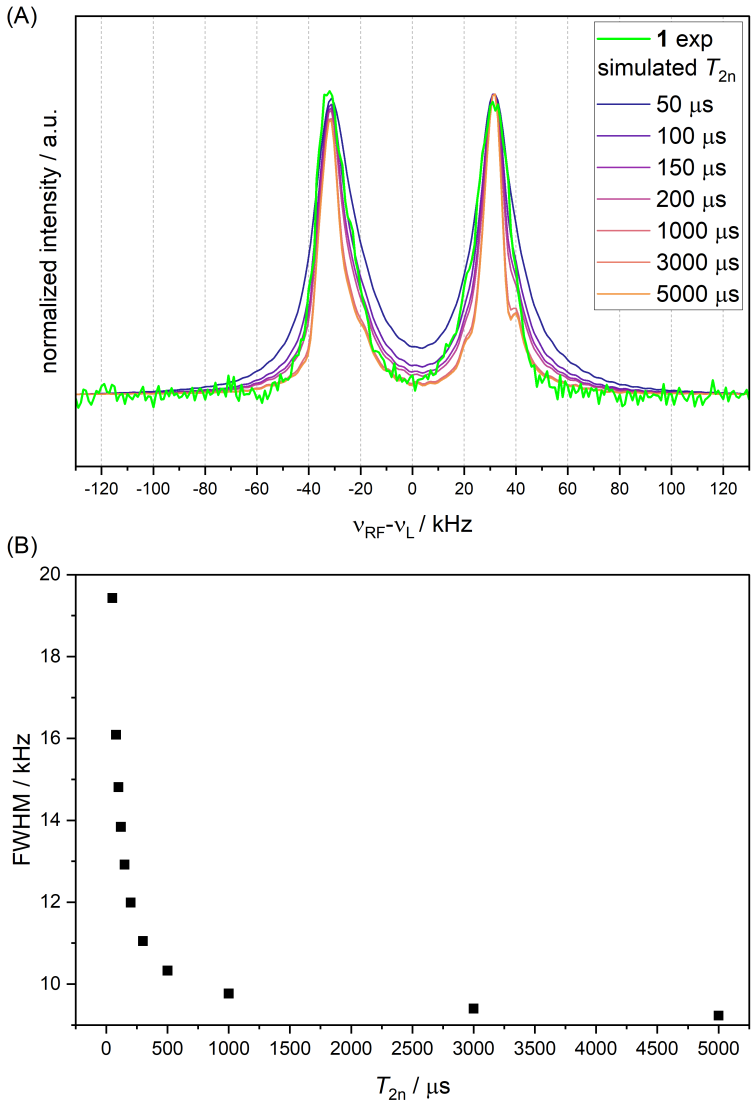

Figure S14: A: Spin dynamics simulations with different T_{2n} relaxation times including the CS and NDC (colored lines) in comparison to the experiment of **1** with t_p(RF)= 200 µs; B: FWHM values of the simulations in A in comparison to the experimental value.

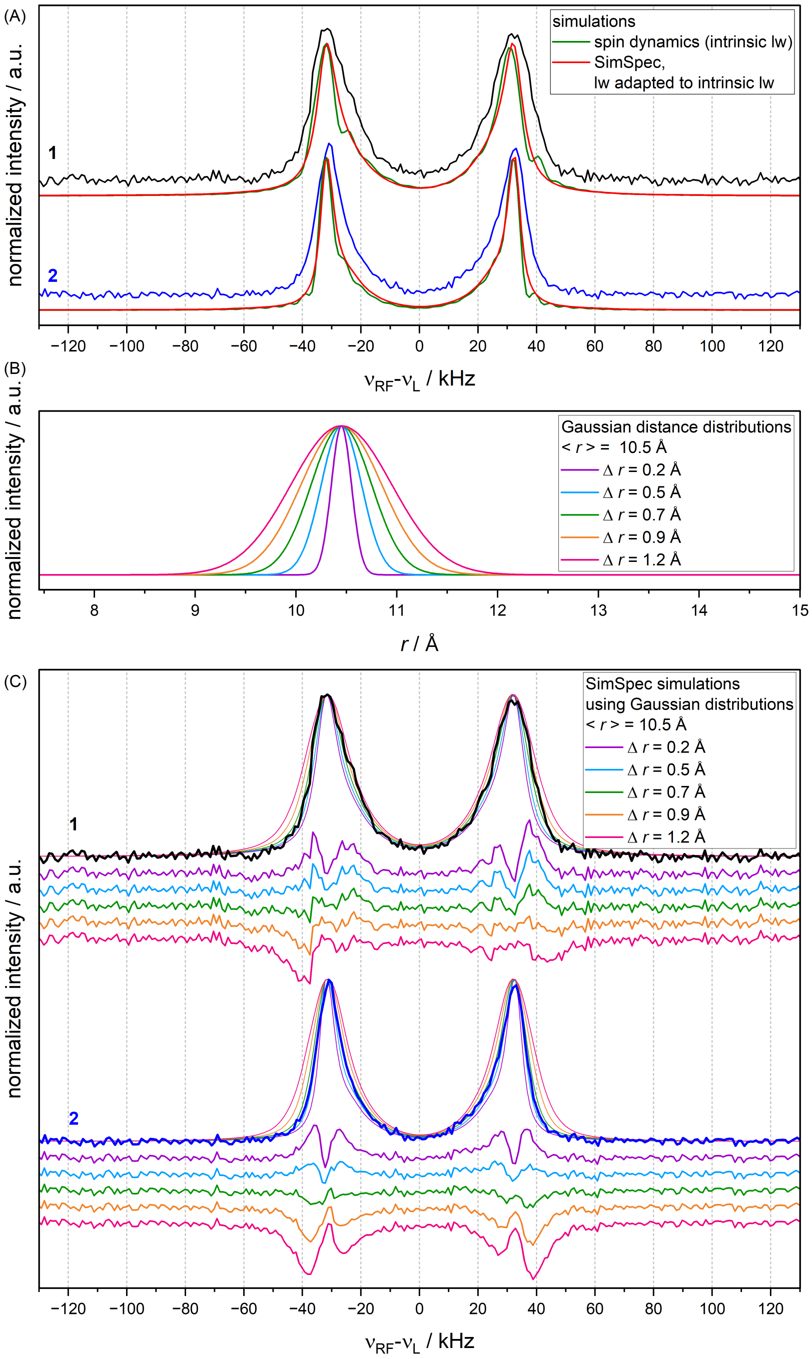

Figure S15: A: estimation of the lw_L parameter (7.3 kHz for **1** and 4.5 kHz for **2**) used for SimSpec simulation based on the spin dynamics simulations; B: tested Gaussian distance distribution; C: SimSpec simulations and residuals using the Gaussian distributions in B.

7 Additional line broadening contributions for the model compounds

To test whether B_0 inhomogeneity contributes significantly to the ENDOR line width, we compared measurements on two different samples of **1** with either ca. 1 cm filling height (i.e. filling out the resonator) or ca. 2 mm. Since the sensitivity is significantly lower for the small sample, we were only able to measure with 100 µs RF pulses (using long RF pulses leads to a loss in sensitivity), which is in a regime where power broadening is no longer the major source of line broadening. The results are summarized in Figure S16 and show that field inhomogeneity can be ruled out as major contribution to the ENDOR line width, as both samples yield practically identical spectra (bottom spectra in Figure S16). This finding agrees with typical field inhomogeneities of 10 ppm (ca. 0.5 kHz for ^{19}F at 1.2 T) [9].

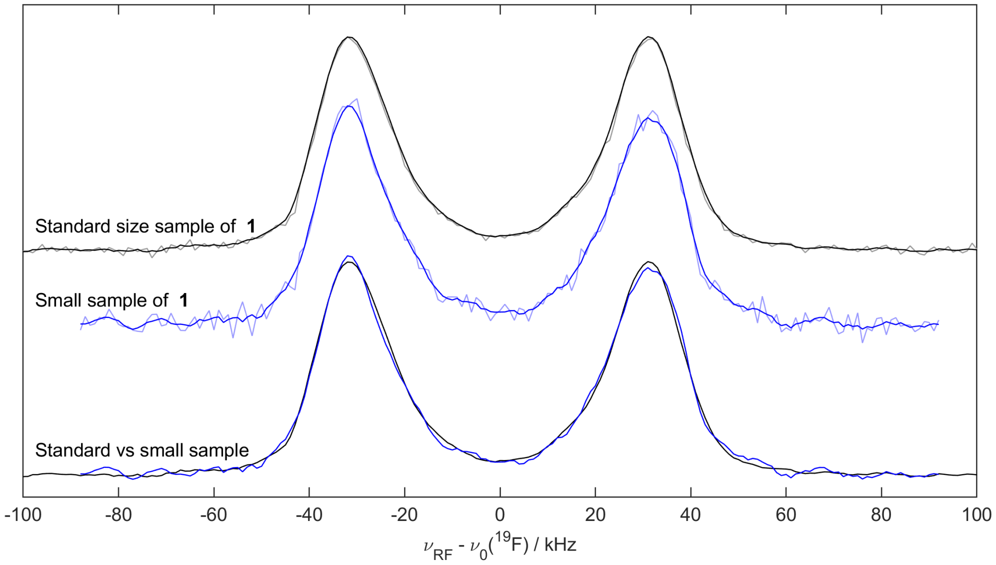

Figure S16: Comparison of ^{19}F ENDOR spectra of **1** measured with 100 µs RF pulses with a standard size sample (black lines, filling height ca. 1 cm) or a smaller sample (blue lines, filling height ca. 2 mm). Because of limited S/N, we compared the data after equal weighting two-point data smoothing as implemented in Xepr. This smoothing did not affect the line width significantly, as can be shown by comparison with the original measurement data, which are depicted semi-transparently in the upper two spectra.

Intermolecular NDC is another contribution to the ENDOR line width, i.e. coupling to solvent nuclei in case of model systems **1** and **2**. Experimentally, we probed these couplings for **2** by repeating our ENDOR measurements in a solvent that contains 50% protonated components, the results of these experiments are shown in Figure S17. Because of significantly faster electronic relaxation, we could not use the longest pulses used in our measurements shown in the main text but were still able to obtain a spectrum with reasonable S/N when using 100 µs RF pulses as done for the test of the B_0 inhomogeneity. Using partially protonated solvents led to an increase in FWHM of ca. 4 kHz. This value is comparable to the line width increase when going from **1** to **2** and is likely mostly caused by protons attached to solvent molecules in the first solvent sphere around the ^{19}F nucleus. The NDCs to solvent nuclei can currently not be included explicitly in ENDOR simulations, since the spatial arrangement of the molecules is not known and because there are too many solvent nuclei present.

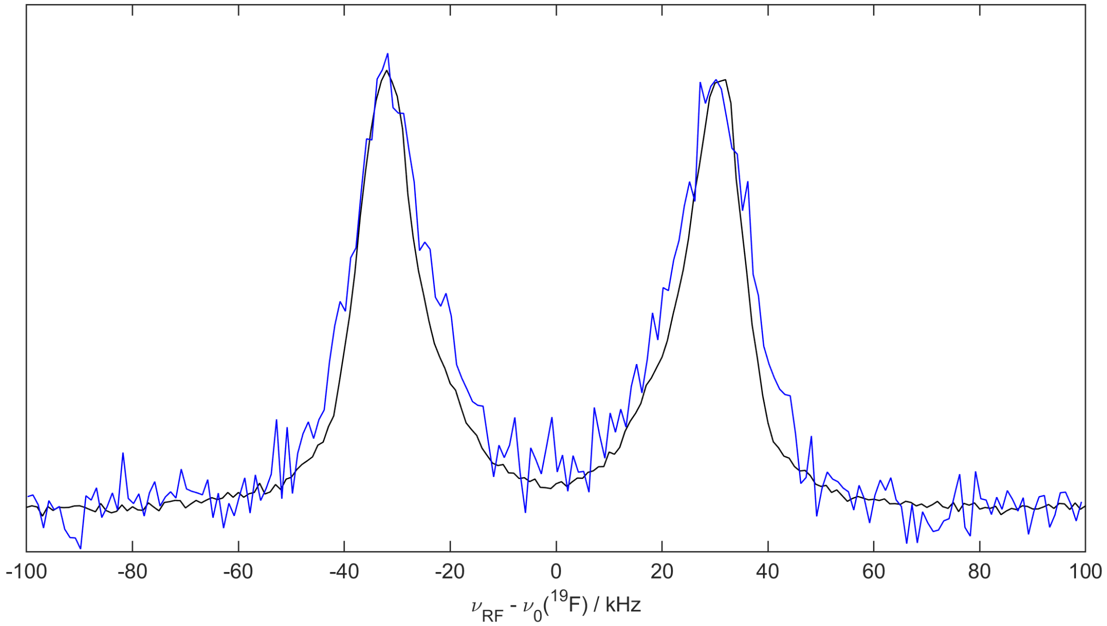

Figure S17: Comparison of ^{19}F ENDOR spectra of **2** measured with 100 μs RF pulses with standard solvent (black lines, DMSO-d_6/CD_3OD, 1:1.5) or with a solvent mixture that contains 50% protonated solvent components (blue lines, DMSO/methanol, 1:1.5, each component as a 50/50 mixture of protonated and fully deuterated version). Because of faster relaxation, the measurements in partially protonated solvent yielded relatively low S/N even after ca. 40 h of averaging.

The deuteration degree of the phenyl ring in **2** was about 90% per proton site, which means that a significant percentage of molecules will carry one proton *ortho* to the fluorine nucleus. To investigate the contributions from such partly deuterated molecules, we performed simulations considering a situation in which 70% of molecules are fully deuterated and 30% only partly deuterated in *ortho* position to ^{19}F. We found only a small additional damping/broadening effect in the time domain simulations.

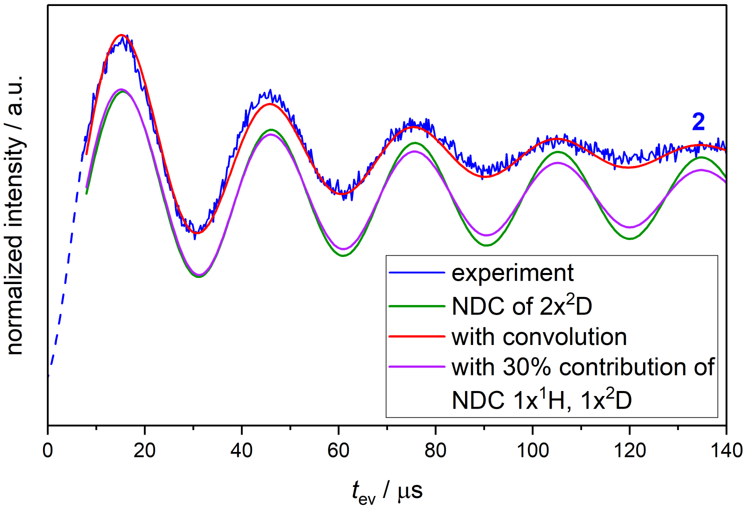

Figure S18: TD ENDOR traces for **2** (blue) with spin dynamics simulation: considering two deuterons (green), 30% contribution of one proton and one deuteron (purple) and convoluted with an additional Lorentzian line broadening of 2.9 kHz (red); blue, green and red line as shown in Figure 5 in the main text.

8 Spin labelling of the RNA and spectroscopic characterization

RNA strand B was bought with a phosphorothioate modification at C3 and spin labelling was performed according to the protocol from Qin *et al.* [10]. Briefly, (4-deutero-1-oxyl-2,2,5,5-tetrakis(trideuteromethyl)-2,5-dihydropyrrol-3-yl)dideuteromethyl methanesulfonate (1 mg, 3.8 µmol) was dissolved in acetone (200 µL) and combined with sodium iodide in acetone (1 M, 3 µL equivalent to 450 µg, 3.0 µmol, 0.75 equivalents). The mixture was incubated at 37 °C for 1 h during which a white precipitate formed. The solution was centrifuged for 10 min at 13000×g and the supernatant was recovered. The precipitate was washed with acetone, centrifuged, and the supernatant was recovered. The supernatants were combined and the solvent was removed using a SpeedVac™ vacuum concentrator to result in 4-deutero-3-(dideuteroiodomethyl)-2,2,5,5-tetrakis(trideuteromethyl)-2,5-dihydropyrrol-1-oxyl.
The iodinated spin label was dissolved in acetonitrile (4 µL). PBS (1 µL 20× PBS) and RNA (500 µM, 15 µL) were added to result in 20 µL of the reaction mixture. It was incubated in the dark at room temperature for about 14 h. The purification of the RNA from excess spin label was performed using the Monarch® RNA Cleanup kit (New England Biolabs, Ipswich, United States). The elution of the RNA was performed using DEPC treated D_2O. The labelling efficiency of the RNA was determined by CW EPR to be 31%. The labelled RNA was freeze-dried and re-dissolved in D_2O to the required concentration. Subsequently, the labelled strand B was combined with strand A (see main text), which was purchased with the fluorine spin label attached.

The echo detected EPR spectrum for the RNA construct is shown in Figure S19 along with a SimSpec simulation. As simulation parameters $g = [2.00825, 2.00595, 2.00211]$, $A_{x,y,z}(^{14}\mathrm{N}) = [10, 17, 104]$ MHz, $P_{x,y,z}(^{14}\mathrm{N}) = [1.2, 0.54, -1.7]$ MHz were used. Observer positions for ^{19}F ENDOR measurements are indicated in the Figure.

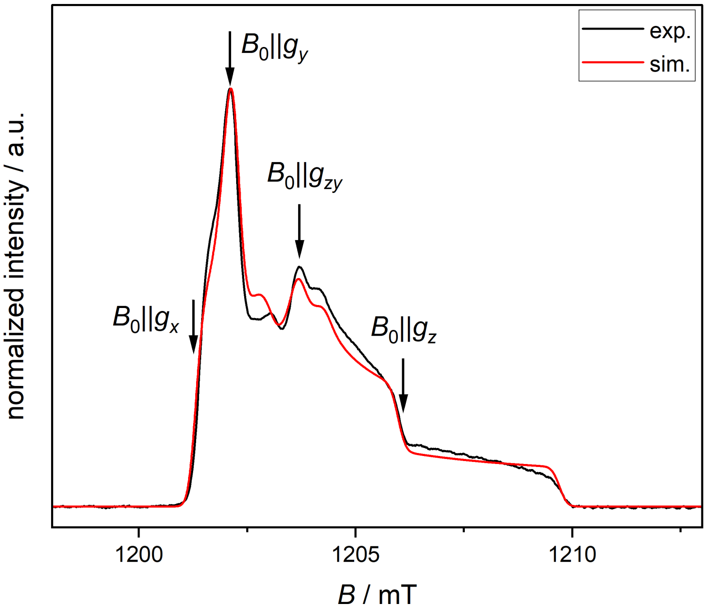

Figure S19: Echo-detected EPR spectrum for the spin labelled RNA construct (black), its simulation (red) and the measurement positions indicated by arrows.

Measurements of T_{1e} and T_M for the delay used in ENDOR experiments of the RNA construct are shown in Figure S20.

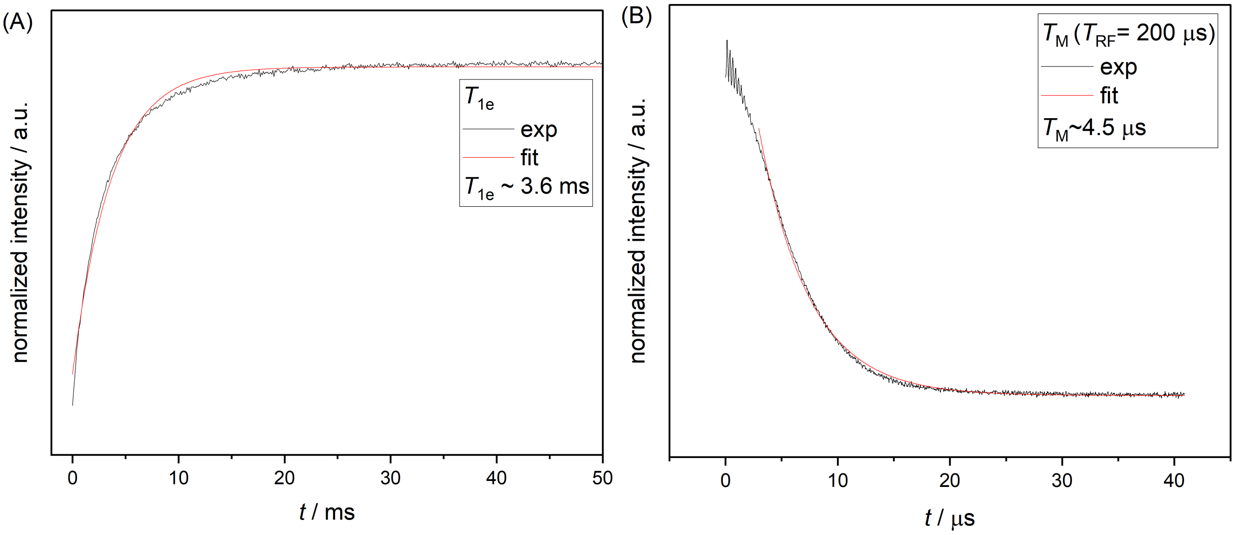

Figure S20: Measurements of T_{1e} (A) and T_M for $T_{RF} = 200$ μs (B) for the RNA construct.

9 Simulations for the RNA Construct

The spin dynamics simulation was performed using g=[2.00825, 2.00595, 2.00211], $A_{x,z,z}(^{14}\mathrm{N})$=[10, 17, 104] MHz, $P_{x,z,z}(^{14}\mathrm{N})$=[1.2, 0.54, −1.7] MHz, $T_{\mathrm{dip}} = 36.4$ kHz and the NDC values given in Table S3. The g-values and A and P tensors for ^{15}N were optimized according to the echo-detected EPR spectrum (see Figure S19). The Euler angles for the NDCs were estimated as mean values from the predicted rotamer clouds as the angles describe the orientation of the interspin vector with respect to the g-tensor of the nitroxide.

Table S3: NDC values for the spin dynamics simulation of the RNA construct.

	$r(\mathrm{F}-\mathrm{H})$ / Å	$D_{\mathrm{dip},1}$ / kHz	α / °	β / °
#1	2.0	14.3	-45	0
#2	2.4	8.3	0	45
#3	2.4	8.3	0	-135

SimSpec simulations with different Gaussian distributions were systematically performed for different lw_{L} parameters. The optimal simulations and parameters for the Gaussian distributions are listed in Figure S21. The RMSDs are plotted in Figure S22 and the optimal values are circled in red.

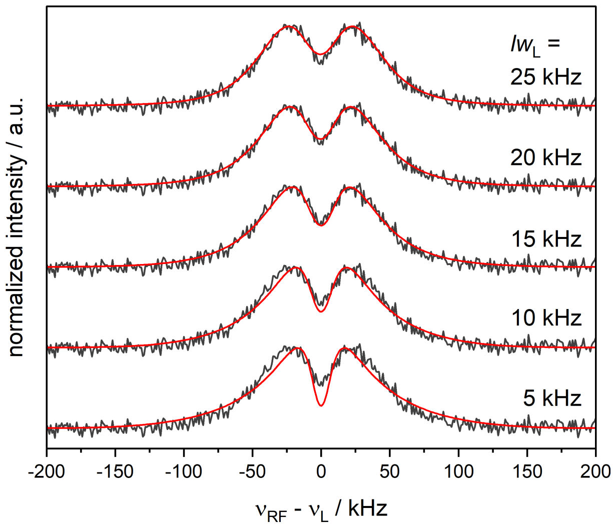

lw_{L} / kHz	<r> / Å	Δr / Å	RMSD / 10^{-3}
5	15.2	7.5	3.45
10	14.7	6.6	2.80
15	13.7	5.2	2.54
18	13.2	4.2	2.45
20	13.2	4.2	2.45
25	12.7	3.3	2.50

Figure S21: Left: SimSpec simulations of the ENDOR spectra with the Gaussian distributions that have the smallest RMSDs for different Lorentzian lw_{L} parameters; Right: values describing the Gaussian distributions.

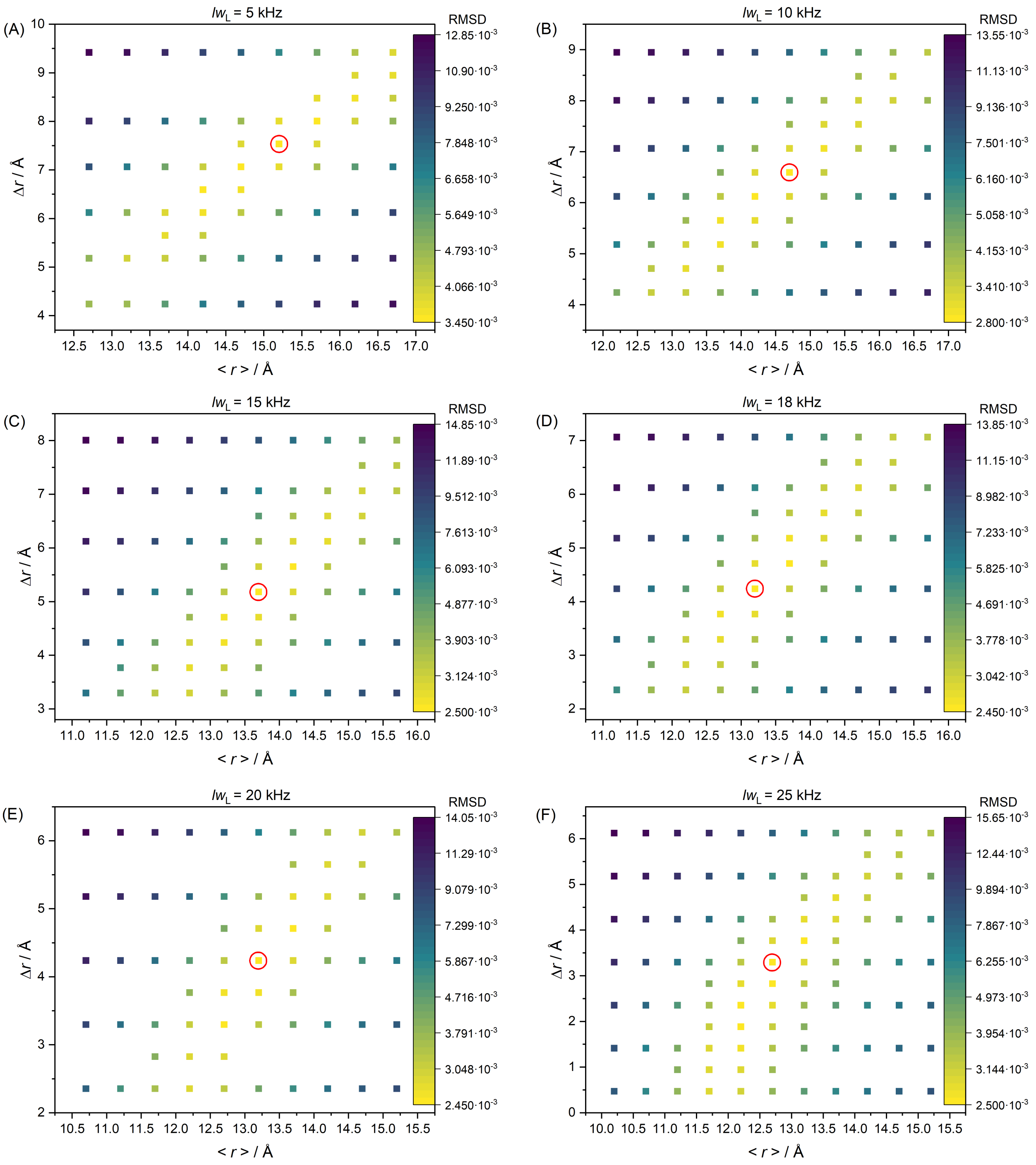

Figure S22: RMSDs between the ENDOR spectrum and SimSpec simulations with unimodal Gaussian distributions as a function of the lw_L parameter and the mean ($<r>$) and the FWHM (Δr); minimal value circled red.

The approximation of the spin dynamics calculation by the SimSpec simulations was tested for different line shapes and line with parameters. The RMSDs are plotted in Figure S23.

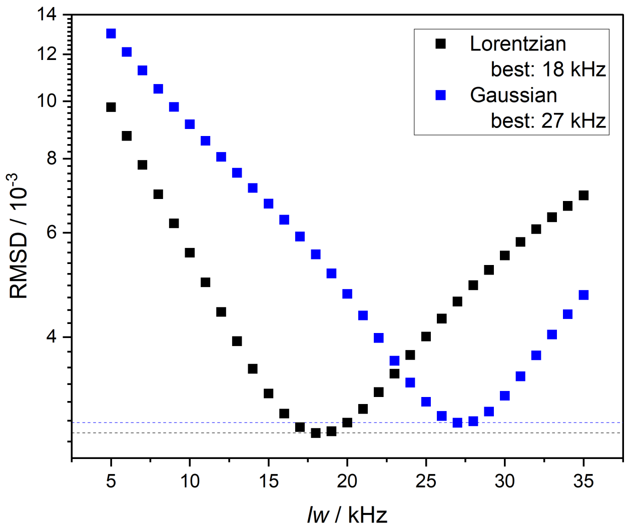

Figure S23: RMSDs between spin dynamics calculation including NDCs and SimSpec simulations with either Lorentzian (black) or Gaussian (blue) line shape convolution as a function of the lw parameter, dotted lines indicate minimal values.

The optimization of the Gaussian distance distribution to match SimSpec simulations with the ENDOR spectra was repeated for $lw_{\mathrm{G}} = 27\,\mathrm{kHz}$. The corresponding RMSDs are shown in Figure S24 (A) and the optimal simulation in panel B.

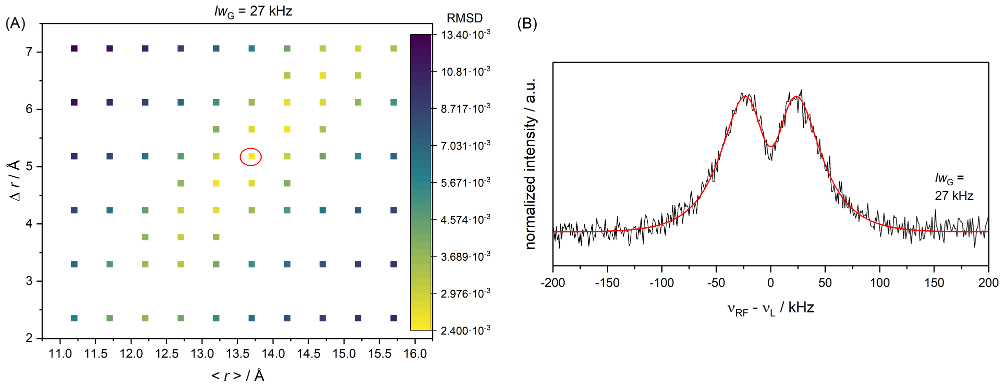

Figure S24: A: RMSDs for the SimSpec simulation of the ENDOR spectrum for different Gaussian distance distributions, optimal value circled in red; B: simulation (red) of the ENDOR spectrum (black) with the optimal distribution ($<r>$=13.7 Å and $\Delta r = 5.2$ Å).

10 Line width parameters reported in literature

In Table S4 lw parameters reported previously in literature are listed.

Reference	lw_{L} / kHz	lw_{G} / kHz	System	considered mechanisms
Meyer *et al.* [6]	/	10-26	nitroxide, model compound	PB
	/	20-44	nitroxide, RNA	PB, flexibility
Kehl *et al.* [11]	20	/	nitroxide, model compound	PB
Asanbaeva *et al.* [12]	(n.s.)		TAM, model compound	PB, nuclear relaxation
Judd *et al.* [13]	30-60	/	Phe-CF_3 group, protein	PB, relaxation, distance distribution
Meyer *et al.* [14]	/	25	tyrosyl, protein	PB
Asanbaeva *et al.* [15]	/	20	TAM, DNA	PB
Schumann *et al.* [16]	/	36/60	Cu, Quadruplexes	(n.s.)
Seal *et al.* [17]	30-35	/	Gd, protein	(n.s.)
Wiechers *et al.* [7]	20	/	nitroxide, model compound	PB
Bogdanov *et al.* [18]	13-42	/	Gd, protein	PB, relaxation, distribution
Bogdanov *et al.* [19]	17-24	/	Gd, protein	PB, relaxation, CS, NDC, distributions
	12-33	/	trityl, protein	
	18-33	0-30	nitroxide, protein	
	2	23	Cu, protein	
	10	/	all systems	for distribution simulations
Gauger *et al.* [20]	(n.s.)		nitroxide, DNA	(n.s.)
Remmel *et al.* [21]	7-20	/	nitroxide, RNA	PB

Table S4: lw parameters reported in literature so far, some parameters have not been specified (n.s.).

11 Analytics of deuterated *p*-fluorophenol, compounds 1 and 2

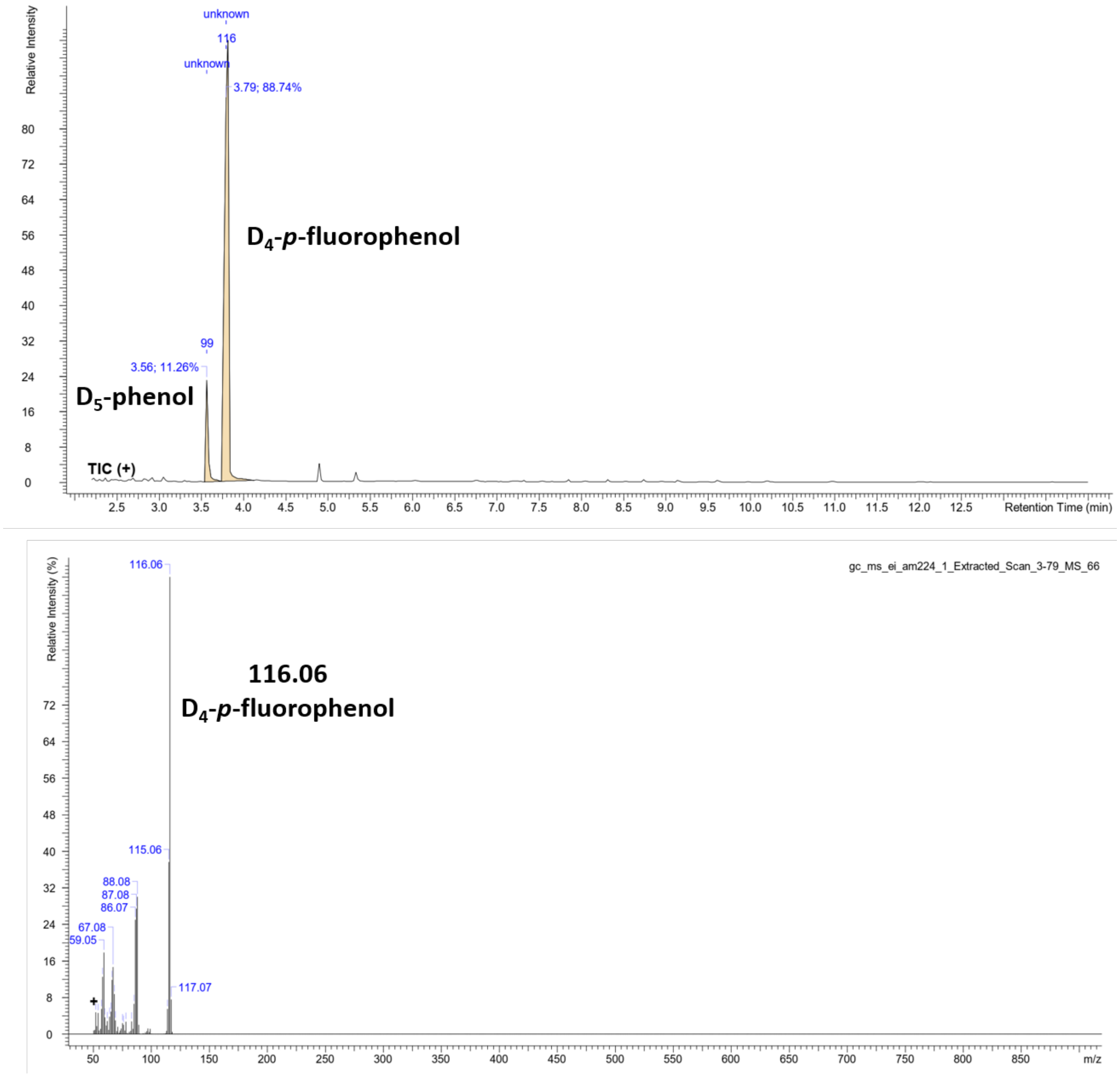

Figure S25: LCMS analytics of deuterated *p*-fluorophenol.

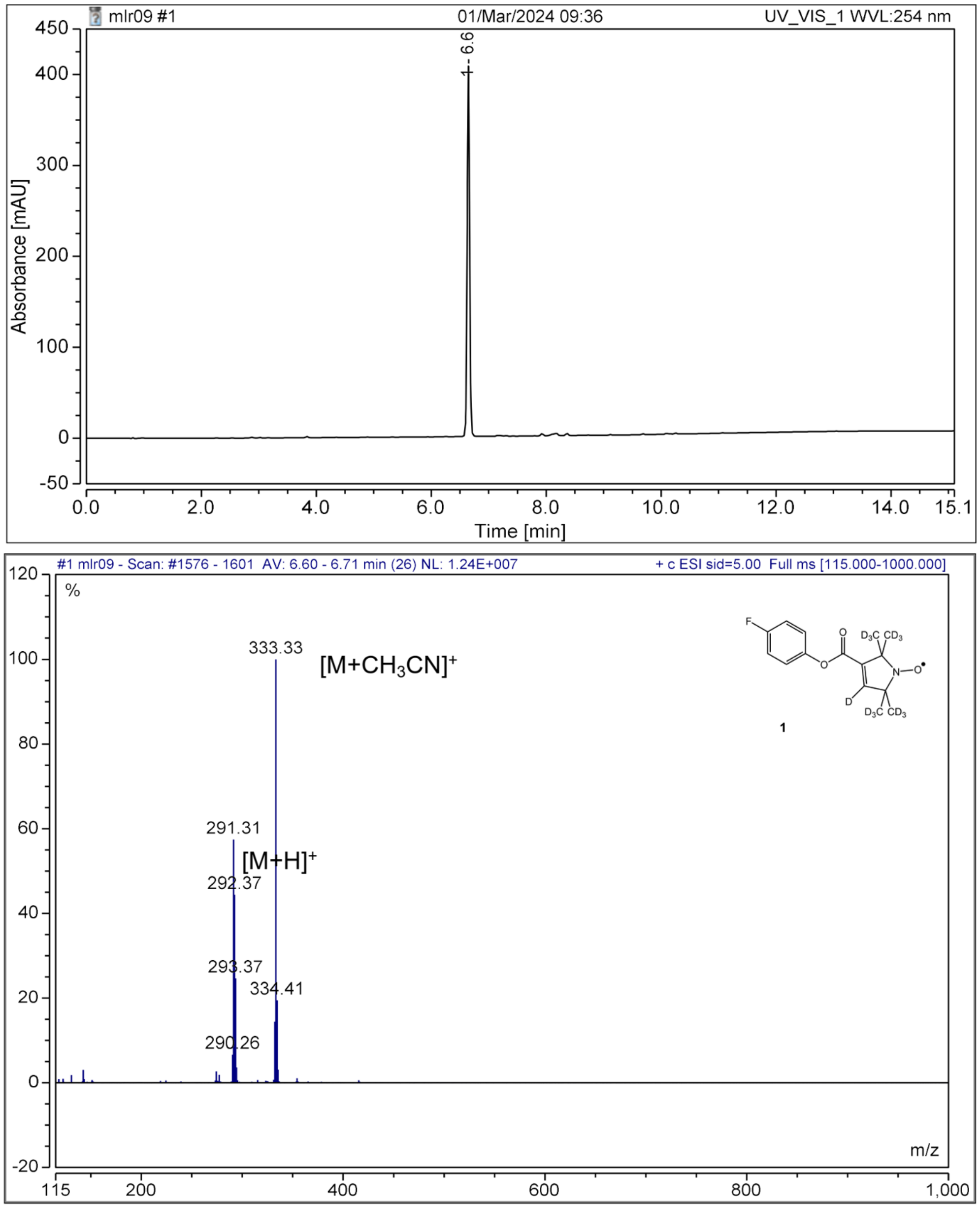

Figure S26: LCMS analytics of **1**.

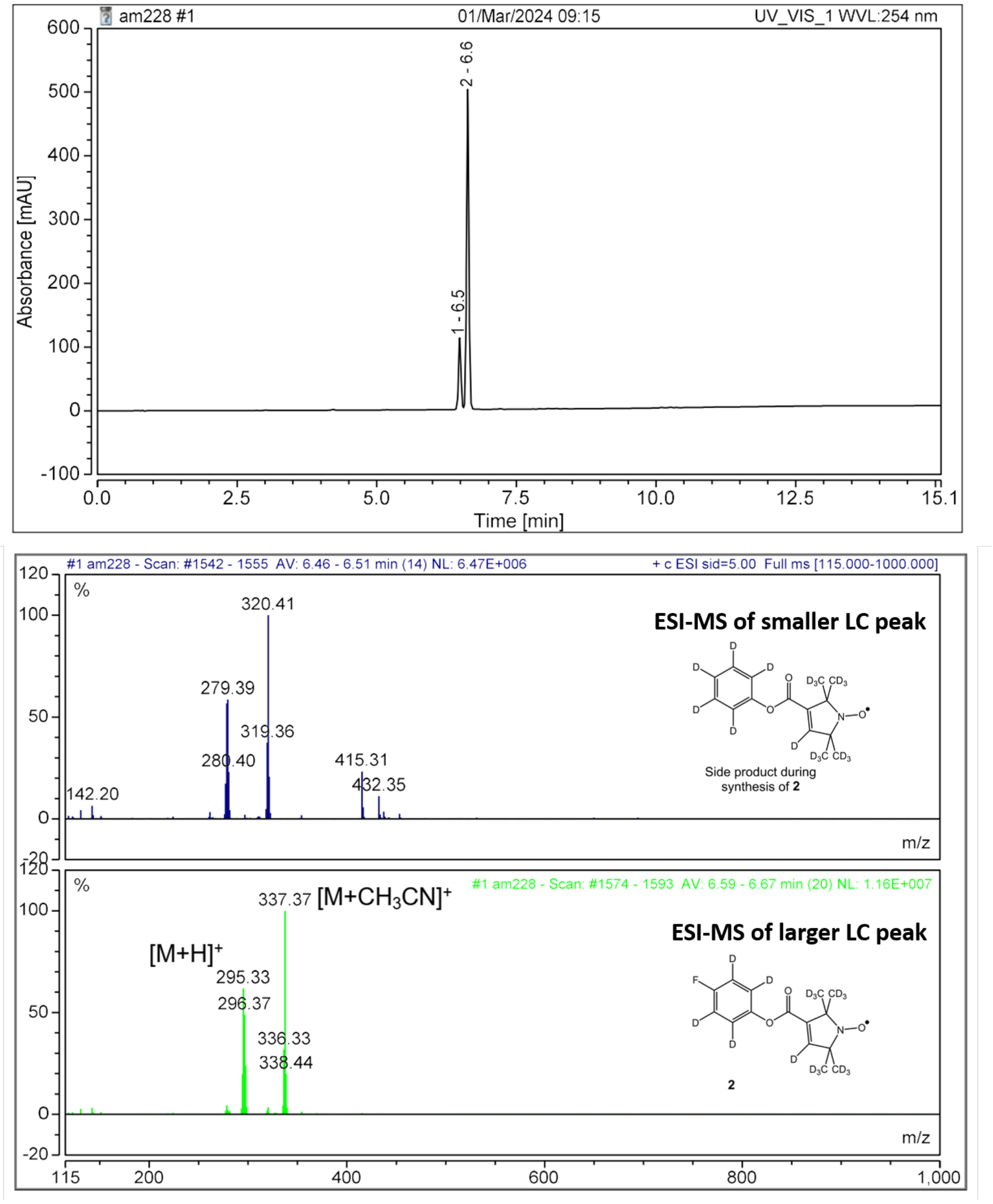

Figure S27: LCMS analytics of **2** including analytics of the side product where defluorohydrogenation has occurred [5].

References

(1) J. Pirrwitz and D. Schwarz, *DDR patent: DD222017A1 (WP C 07 D/260 901 6), German Democratic Republic*, 1984.

(2) L. A. Shundrin, I. A. Kirilyuk and I. A. Grigor'ev, *Mendeleev Communications*, 2014, **24**, 298–300.

(3) M. M. Haugland, A. H. El-Sagheer, R. J. Porter, J. Pena, T. Brown, E. A. Anderson and J. E. Lovett, *Journal of the American Chemical Society*, 2016, **138**, 9069–9072.

(4) G. Úr, T. Kalai and K. Hideg, *Tetrahedron Letters*, 2016, **57**, 778–780.

(5) Y. Sawama, A. Nakano, T. Matsuda, T. Kawajiri, T. Yamada and H. Sajiki, *Organic Process Research & Development*, 2019, **23**, 648–653.

(6) A. Meyer, S. Dechert, S. Dey, C. Höbartner and M. Bennati, *Angewandte Chemie International Edition*, 2020, **59**, 373–379.

(7) H. Wiechers, A. Kehl, M. Hiller, B. Eltzner, S. Huckemann, A. Meyer, I. Tkach, M. Bennati and Y. Pokern, *Journal of Magnetic Resonance*, 2023, 107491.

(8) A. Schweiger and G. Jeschke, *Principles of pulse electron paramagnetic resonance*, Oxford University Press on Demand, 2001.

(9) *EPR Spectroscopy: Fundamentals and Methods*, ed. D. Goldfarb and S. Stoll, Wiley, 2018.

(10) P. Z. Qin, I. S. Haworth, Q. Cai, A. K. Kusnetzow, G. P. G. Grant, E. A. Price, G. Z. Sowa, A. Popova, B. Herreros and H. He, *Nature Protocols*, 2007, **2**, 2354–2365.

(11) A. Kehl, M. Hiller, F. Hecker, I. Tkach, S. Dechert, M. Bennati and A. Meyer, *Journal of Magnetic Resonance*, 2021, **333**, 107091.

(12) N. Asanbaeva, A. Sukhanov, A. Diveikina, O. Rogozhnikova, D. Trukhin, V. Tormyshev, A. Chubarov, A. Maryasov, A. Genaev, A. Shernyukov et al., *Physical Chemistry Chemical Physics*, 2022, **24**, 5982–6001.

(13) M. Judd, E. H. Abdelkader, M. Qi, J. R. Harmer, T. Huber, A. Godt, A. Savitsky, G. Otting and N. Cox, *Physical Chemistry Chemical Physics*, 2022, **24**, 25214–25226.

(14) A. Meyer, A. Kehl, C. Cui, F. A. Reichardt, F. Hecker, L.-M. Funk, M. K. Ghosh, K.-T. Pan, H. Urlaub, K. Tittmann et al., *Journal of the American Chemical Society*, 2022, **144**, 11270–11282.

(15) N. Asanbaeva, D. S. Novopashina, O. Y. Rogozhnikova, V. M. Tormyshev, A. Kehl, A. Sukhanov, A. V. Shernyukov, A. Genaev, A. A. Lomzov, M. Bennati et al., *Physical Chemistry Chemical Physics*, 2023, **25**, 23454–23466.

(16) S. L. Schumann, S. Kotnig, Y. Kutin, M. Drosou, L. M. Stratmann, Y. Streltsova, A. Schnegg, D. A. Pantazis, G. H. Clever and M. Kasanmascheff, *Chemistry–A European Journal*, 2023, **29**, e202302527.

(17) M. Seal, W. Zhu, A. Dalaloyan, A. Feintuch, A. Bogdanov, V. Frydman, X.-C. Su, A. M. Gronenborn and D. Goldfarb, *Angewandte Chemie*, 2023, **62**, e202218780.

(18) A. Bogdanov, V. Frydman, M. Seal, L. Rapatskiy, A. Schnegg, W. Zhu, M. Iron, A. M. Gronenborn and D. Goldfarb, *Journal of the American Chemical Society*, 2024, **146**, 6157–6167.

(19) A. Bogdanov, L. Gao, A. Dalaloyan, W. Zhu, M. Seal, X.-C. Su, V. Frydman, Y. Liu, A. M. Gronenborn and D. Goldfarb, *Phys. Chem. Chem. Phys.*, 2024, **26**, 26921–26932.

(20) M. Gauger, M. Heinz, A.-L. J. Halbritter, L. S. Stelzl, N. Erlenbach, G. Hummer, S. T. Sigurdsson and T. F. Prisner, *Angewandte Chemie International Edition*, 2024, **63**, e202402498.

(21) L. Remmel, A. Meyer, K. Ackermann, G. Hagelueken, M. Bennati and B. E. Bode, *Angewandte Chemie International Edition*, 2024, e202411241.
